# Interleukins in COVID-19 and SARS-CoV-2 Variants: Immunopathogenesis, Therapeutic Perspectives and Vaccine-Induced Immune Responses

**DOI:** 10.3390/ijms27031391

**Published:** 2026-01-30

**Authors:** Supriya Mahajan, Saurabh Mahajan, Akanksha Gusain

**Affiliations:** 1 Department of Microbiology, School of Medical Sciences and Research, Sharda University, Greater Noida 201306, UP, India; 2 Numed Super Speciality Hospital, Greater Noida 201306, UP, India

**Keywords:** interleukins, COVID-19, SARS-CoV-2 variants

## Abstract

The Coronavirus disease 2019 (COVID-19), caused by severe acute respiratory syndrome coronavirus 2 (SARS-CoV-2), is characterized by profound immune dysregulation where interleukins play a central role in determining disease severity and response to interventions. This review summarizes the role of interleukins in the immunopathogenesis of COVID-19, with particular emphasis on differences observed across major SARS-CoV-2 variants. Pro-inflammatory interleukins like IL-1β, IL-6, IL-2, IL-17 and IL-18 are critically involved in cytokine storm, hyperinflammation, and acute respiratory distress syndrome, whereas anti-inflammatory cytokines like IL-10 contribute to immune regulation and resolution of inflammation. Elevated levels of IL-1α, IL-1β, IL-4, IL-8, IL-9, IL-16, IL-18 have been documented in the Delta variant as compared with the Omicron variant, with IL-6 being the most frequent interleukin reported to be increased across all SARS-CoV-2 variants relative to the ancestral Wuhan strain. Elevated IL-2, IL-4, IL-6, and IL-10 levels have been associated with Omicron sub-variants. The review encompasses interleukin-based therapeutic strategies, where several IL-1 and IL-6 inhibitors were studied across clinical trials, but only tocilizumab has shown some promise against severe COVID-19. IL-2, IL-6, IL-15 and IL-21 levels were positively correlated with IgG and neutralizing antibody activity after vaccination with longevity of post-vaccination immunity being determined by IL-2 and IL-7.

## 1. Introduction

The immune system plays a significant role in the pathogenesis of COVID-19, with the innate immune system being the first line of defense initiated by the recognition of pathogen-associated molecular patterns (PAMPs) by pattern recognition receptors (PRRs) expressed on the surface of immune cells, leading to the production of cytokines and other immune mediators [1,2]. Interleukins (ILs) are types of cytokines which can be classified as either pro-inflammatory or anti-inflammatory depending on their effects on the immune system. Pro-inflammatory interleukins, such as interleukin-1β (IL-1β), IL-6, IL-2, IL-7, IL-8, IL-12, IL-17, promote fever, inflammation, macrophage activation and even cytokine storm in COVID-19, leading to severe tissue damage and organ dysfunction. In contrast, anti-inflammatory interleukins, such as IL-10, IL-4, IL-13, IL-37, IL-38 suppress pro-inflammatory cytokines, inhibit Th1-mediated inflammation and have protective effects against cytokine storm as well as hyperinflammation in COVID-19. However, an excessive anti-inflammatory response can lead to immune suppression and impaired viral clearance [3]. This review aims at elaborating the roles of various interleukins in the pathogenesis, severity, treatment and response to vaccination in COVID-19 cases as well as to evaluate their roles in severe acute respiratory syndrome coronavirus 2 (SARS-CoV-2) variants.

## 2. Methods

### 2.1. Search Strategy

We conducted a systematic literature search covering the role of ILs in COVID-19 and SARS-CoV-2 variants with special emphasis on immunopathogenesis, therapeutic perspectives and vaccine-induced immune responses in the following electronic databases: PubMed/MEDLINE, Scopus, Embase and Web of Science. Additional searches were performed in Cochrane Library and Google Scholar for grey literature. The literature search encompassed studies published between August 2002 and October 2025. We included all relevant studies published in peer reviewed journals. Search strategies combined keywords and MeSH terms relating to SARS-CoV-2 and interleukins which included the following: (“SARS-CoV-2” OR “COVID-19” OR “novel coronavirus”); (“interleukin*” OR “IL-6” OR “IL-1β” OR “IL-10” OR “cytokine*”); (“variant*” OR “lineage” OR “mutation”); (“severity” OR “immune response” OR “clinical outcomes”). These terms were used in combinations with Boolean operators (AND/OR) to ensure sensitivity.

### 2.2. Study Eligibility

Original human clinical studies were included reporting roles of different ILs in confirmed SARS-CoV-2 infection. Also included were studies providing association of ILs in SARS-CoV-2 variants (e.g., Alpha, Delta, Omicron) with specific inclusion of Omicron sub-variants. Search was focused on studies showing comparison of Delta vs. Omicron vs. wild type virus in relation to ILs and/or their combinations. The types of studies included observational, cohort, case–control, randomized controlled trials (RCTs), case reports, systematic reviews and meta-analyses. Non-COVID-19 cytokine studies were excluded unless directly informing SARS-CoV-2 biology.

### 2.3. Criteria for SARS-CoV-2 Variant Attribution

Variant attribution across the included studies was based on genomic sequencing, epidemiological context, or authoritative classification systems, depending on study design and data availability. Preference was given to sequence-validated variant data.

### 2.4. Handling of Treatment Era and Evolving Standard of Care

The treatment era in which each study was conducted was systematically considered during data extraction and synthesis, given the rapid evolution of COVID-19 therapeutics and its direct impact on IL-targeted interventions. Studies were interpreted in the context of the prevailing standard of care, regulatory status, and clinical evidence available at the time of patient enrollment.

Studies conducted during the early pandemic and pre-guideline phase (early-mid 2020) primarily evaluated cytokine-targeted therapies—including IL-1 inhibitors (e.g., anakinra, canakinumab), IL-6 pathway inhibitors (e.g., tocilizumab), and JAK inhibitors (e.g., baricitinib)—in the absence of standardized background therapy such as routine corticosteroid use or uniform respiratory support protocols. Such studies were analyzed as exploratory and interpreted with caution due to evolving supportive care practices.

During the evidence accumulation phase (late 2020–2021), increasing clinical trial data and systematic reviews evaluated the efficacy and safety of IL-1 and IL-6 blockade alongside emerging standards of care, including corticosteroids use and structured respiratory support. During this period, multiple observational studies, randomized trials, and systematic reviews evaluated IL-1 and IL-6 blockade with increasing methodological rigor, often alongside evolving supportive care protocols. Studies from this period frequently incorporated structured inflammatory biomarkers and outcome measures, allowing more consistent comparison, although variability in background therapy remained.

Studies conducted during the guideline-informed and regulatory phase (2022 onward) were analyzed separately, particularly those evaluating IL-6 inhibitors, following their inclusion in international treatment recommendations and the WHO prequalification of tocilizumab. Clinical trials and observational studies from this era generally reflect combination therapy with corticosteroids and, in some cases, antivirals and anticoagulation, necessitating careful attribution of clinical benefit to cytokine blockade alone.

For emerging or less-established IL targets, including the IL-17/Th17 axis and IL-22-related pathways, evidence was derived largely from mechanistic studies, pilot clinical trials, and immunophenotyping analyses conducted across different treatment eras. These studies were interpreted within the context of contemporaneous standard-of-care therapies and disease severity definitions, recognizing the absence of guideline endorsement for these targets during most study periods.

No studies were excluded solely on the basis of treatment era. Instead, treatment era was incorporated as an analytical variable during narrative synthesis to account for heterogeneity in therapeutic efficacy, biomarker dynamics, and clinical outcomes across studies evaluating anti-interleukin strategies in COVID-19.

### 2.5. Handling of Vaccination Status

Vaccination status was systematically incorporated during study selection and synthesis to account for vaccine-induced interleukin responses. Studies were stratified by mRNA vaccine type, vaccination regimen (primary series versus booster doses), and participant immune status, including healthy adults, elderly individuals, and immunocompromised populations.

Longitudinal studies assessing spike-specific cellular immune responses were prioritized to capture temporal changes in pro- and anti-inflammatory interleukin profiles following vaccination. Evidence on durable T-cell responses and cytokine signatures associated with effective vaccine-induced immunity was analyzed to distinguish vaccination-related responses from those following natural infection.

Interleukin involvement in vaccine-induced B-cell immunity was evaluated separately, particularly in immunocompromised cohorts.

### 2.6. Approach to Synthesis

Due to substantial heterogeneity across included studies with respect to study design, patient populations, interleukin measurement methods, clinical severity definitions, and circulating SARS-CoV-2 variants, a qualitative narrative synthesis was performed rather than a quantitative meta-analysis.

Findings were summarized descriptively and organized according to individual ILs, clinical severity, and SARS-CoV-2 variant or variant-dominant period. Where available, consistent patterns across multiple studies were highlighted, and discordant findings were interpreted in the context of differences in study design, sample size, timing of cytokine measurement, vaccination status, and therapeutic interventions.

Formal evidence grading systems were not applied due to substantial heterogeneity across study designs, variant attribution methods, and interleukin measurement approaches; findings were therefore synthesized narratively.

## 3. IL-1 in COVID-19

COVID-19-induced epithelial injury leads to the secretion of IL-1α and IL-1β, which further promote the recruitment of neutrophils and monocytes to the site of infection [4]. Studies have also shown elevated levels of IL-1β and interleukin-1 receptor antagonist (IL-1Ra) in the peripheral blood and bronchoalveolar lavage fluid (BALF) of patients with COVID-19-induced pneumonia [5]. IL-1β is activated and released following activation of the inflammasome that constitutes an important arm of the innate immunity [6].

### 3.1. Mechanisms of IL-1 in COVID-19 Pathogenesis

#### 3.1.1. Auto-Inflammatory Loop Causing Cytokine Cascade

**Figure 1:**
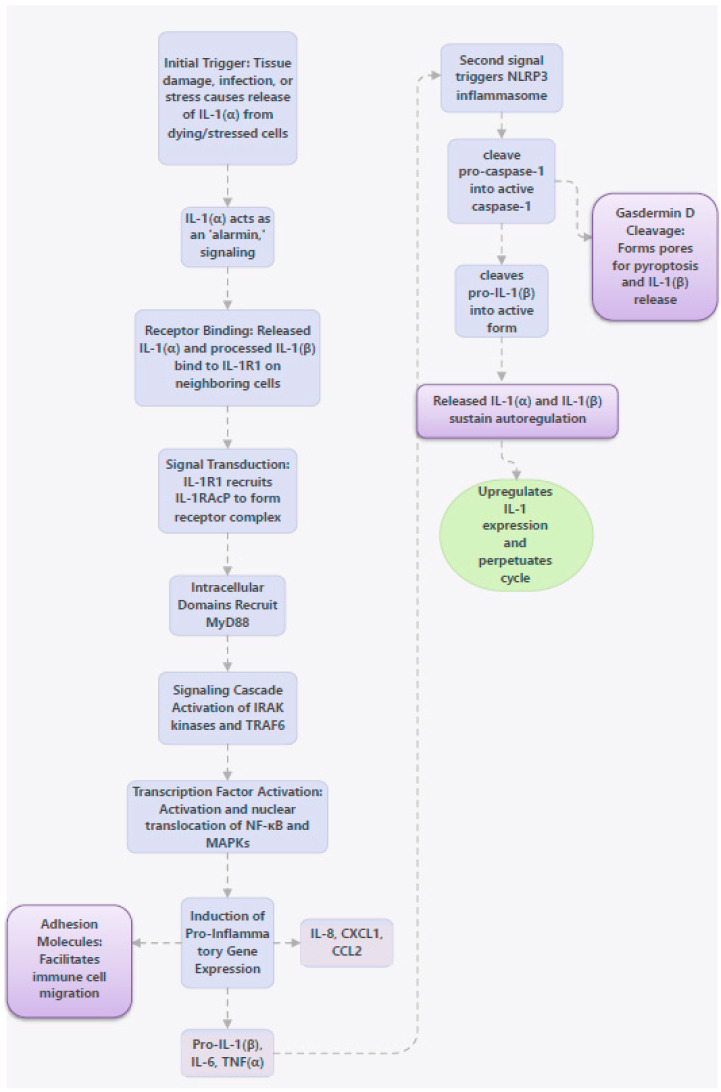
IL-1-driven autoinflammatory loop underlying cytokine cascade.

IL-1 exhibits a striking property of the auto-inflammatory loop wherein it triggers the activation of nuclear factor kappa-light-chain-enhancer of activated B cells (NF-κB) and mitogen-activated protein kinase (MAPK) pathways, which further lead to the production of more IL-1, other pro-inflammatory cytokines (e.g., IL-6, TNF-α), as well as chemokines that recruit neutrophils and monocytes. Recruited immune cells further release IL-1β, often after inflammasome activation, thereby reinforcing the inflammatory response. COVID-19 causes dysregulation of this loop, resulting in excessive inflammation and cytokine storm syndromes [7,8]. Figure 1 depicts the mechanism of IL-1-induced auto-inflammatory loop causing cytokine cascade. Initial Trigger (Alarmin Function): The loop is often initiated by tissue damage, infection, or stress, leading to the release of pre-formed, biologically active IL-1α from dying or stressed cells (e.g., epithelial cells, keratinocytes). IL-1α acts as an “alarmin,” signaling danger to nearby cells. Receptor Binding and Signal Transduction: The released IL-1α and, subsequently, processed IL-1β bind to the interleukin-1 receptor type 1 (IL-1R1) on neighboring cells, such as resident macrophages, endothelial cells, and fibroblasts. Intracellular Signaling Cascade: Upon binding, IL-1R1 recruits an accessory protein (IL-1RAcP) to form a complete receptor complex. The intracellular domains of these receptors (TIR domains) then recruit the adaptor protein MyD88. This initiates a signaling cascade involving IRAK kinases and TRAF6, culminating in the activation and nuclear translocation of key transcription factors, primarily nuclear factor kappa-light-chain enhancer of activated B cells (NFkB) and mitogen-activated protein kinases (MAPKs). Pro-Inflammatory Gene Expression: NFkB and MAPKs enter the nucleus and induce the transcription of a broad range of proinflammatory genes. These include genes for the following: 1. Cytokines, including IL-1β, IL-6, and TNFα. 2. Chemokines, including IL-8, CXCL1, and CCL2, which act as powerful chemoattractants for other immune cells, particularly neutrophils and monocytes. 3. Adhesion molecules, which facilitate the migration of immune cells from the bloodstream into the inflamed tissue. Inflammasome Activation and Amplification: The newly produced pro-IL-1β accumulates in the cell cytosol. A second signal (e.g., ATP release from damaged cells, microbial components) triggers the assembly of multi-protein complexes called inflammasomes (e.g., NLRP3 inflammasome). Caspase-1 Activity and IL-1β Release: Activated inflammasomes cleave pro-caspase-1 into active caspase-1. Caspase-1, in turn, cleaves the inactive pro-IL-1β into its mature, highly active form. Caspase-1 also cleaves Gasdermin D (GSDMD), which forms pores in the cell membrane, leading to a lytic form of cell death (pyroptosis) and the release of mature IL-1β into the extracellular space. Sustained Autoregulation: The released IL-1α and IL-1β act on the same cells (autocrine signaling) and surrounding cells (paracrine signaling), further upregulating IL-1 expression and perpetuating the entire cycle [7,8].

#### 3.1.2. Pyroptosis

Another interesting feature related to IL-1 is that, in addition to triggering a cytokine storm, SARS-CoV-2 can induce pyroptosis which is an inflammatory form of programmed cell death characterized by the activation of pro-inflammatory signaling pathways. A hallmark of pyroptosis is caspase-1 activation, which enables affected cells to release increased levels of IL-1β and IL-18 [9].

#### 3.1.3. Endothelial Dysfunction and Coagulopathy

IL-1β activates endothelial cells, increasing vascular permeability and expression of adhesion molecules. This contributes to endothelial injury, microvascular inflammation, and the hypercoagulable state frequently observed in severe COVID-19 cases [10].

#### 3.1.4. Contribution to Lung Injury and ARDS

In the lungs, IL-1β disrupts the alveolar–capillary barrier, enhances neutrophil-mediated injury, and promotes fibroblast activation, thus contributing to pulmonary edema, hypoxemia, and progression to acute respiratory distress syndrome (ARDS) [11,12].

## 4. IL-2 in COVID-19

Most studies have shown that IL-2 is not significantly altered in severe COVID-19 patients. A study demonstrated that significantly higher IL-2 levels were induced in patients with asymptomatic or mild COVID-19 disease [13]. A systematic review also demonstrated that, despite elevated IL-2 levels in COVID-19 patients, no association with severe disease was observed [14]. In contrast, another study showed that raised IL-2 disrupted the blood–brain barrier in two COVID-19 patients with brain tumor by activating the microglia and an exacerbated immune response [11].

### 4.1. Mechanisms of IL-2 in COVID-19 Pathogenesis

#### 4.1.1. Effects on Regulatory T Cells and Immune Balance

IL-2 is a key growth factor for T cells, promoting clonal expansion, survival, and differentiation of effector T lymphocytes. It is also essential for the maintenance and function of regulatory T cells (Tregs), which suppress excessive immune responses. Dysregulated IL-2 signaling in COVID-19 may impair Treg homeostasis, leading to reduced immune regulation and enhanced inflammatory damage, particularly in severe and critical cases [15].

An interesting study [16] showed that IL-2 was raised in severe patients but decreased in critical patients with COVID-19 pneumonia. The authors claimed that since IL-2, also known as the T cell growth factor, is mainly produced by activated CD4+ and CD8+ T cells; hence IL-2 at low concentration inhibits CD4+ and CD8+ T cell activation by maintaining the activity and survival of T- regulatory cells. IL-2 receptor (IL-2R) expression in peripheral blood mononuclear cell (PBMC) and CD8+ T cell count were significantly lower in critical COVID-19 patients. Therefore, a decrease of CD8+ T cells in critical COVID-19 patients may be related to the inhibition of the IL-2 signaling pathway.

#### 4.1.2. Role in Cytokine Release Syndrome (CRS)

**Figure 2:**
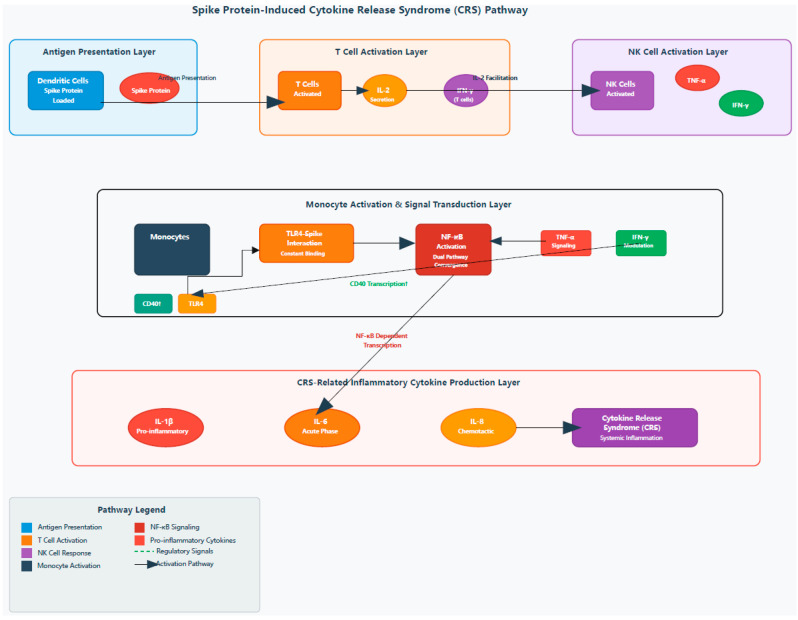
IL-2–spike protein synergy driving cytokine release syndrome.

Spike protein and IL-2 synergistically stimulate PBMCs to produce cytokine release syndrome (CRS)-associated inflammatory factors, possibly mediated by monocytes, as shown in Figure 2. Dendritic cells (DCs) loaded with the spike protein activate T cells to secrete IL-2, which subsequently promotes the production of TNF-α and IFN-γ by natural killer (NK) cells and IFN-γ by T cells. IFN-γ further enhances the transcription of CD40, facilitating the stable localization of TLR4 on the monocyte membrane surface, resulting in sustained interaction between the spike protein and TLR4 and leading to the activation of NF-κB signaling. TNF-α also activates NF-κB in monocytes, which, together with IFN-γ, modulates the NF-κB-dependent transcription of CRS-related inflammatory cytokines, including IL-1β, IL-6, and IL-8 [17,18].

## 5. IL-6 in COVID-19

IL-6 is not only a central mediator of CRS, but also facilitates in the diagnosis of sepsis by inducing the production of C-reactive protein (CRP) and procalcitonin (PCT) [19]. Several studies have reported elevated levels of IL-6 in severe and critically ill COVID-19 patients [20,21,22,23]. A study demonstrated that IL-6 levels remained at moderate concentrations during the first 5 days after admission in severe patients who survived or did not require mechanical ventilation, whereas persistently high IL-6 levels were observed throughout the disease course in patients who either died or developed respiratory failure. Additionally, the IL-6-to-lymphocyte ratio showed a positive correlation with mortality risk in COVID-19 patients [24].

### 5.1. Mechanisms of IL-6 in COVID-19 Pathogenesis

#### 5.1.1. Triggering and Amplification of Inflammation

IL-6 is produced by multiple cell types, including macrophages, monocytes, T cells, endothelial cells and fibroblasts, in response to SARS-CoV-2 infection. Viral entry through ACE2 and the subsequent epithelial cell damage activate innate immune sensors (e.g., Toll-like receptors), leading to NF-κB activation and robust IL-6 production, along with other pro-inflammatory cytokines (e.g., TNF-α, IL-1β). This contributes to a positive feedback loop of inflammation that can escalate into a cytokine release syndrome (CRS) in severe COVID-19 [19,25].

#### 5.1.2. IL-6 Signaling Pathways

IL-6 exerts its effects via two major signaling routes. First is the classical (cis) signaling route where IL-6 binds to membrane-bound IL-6 receptor (mIL-6R) and gp130 on immune cells, generally associated with protective and regulatory responses. Second is the trans-signaling route, where IL-6 binds soluble IL-6R (sIL-6R) and gp130 on many cell types, including endothelial and epithelial cells not expressing mIL-6R. This pathway is pro-inflammatory and expands the range of cells that respond to IL-6, promoting vascular permeability, leukocyte recruitment, and tissue inflammation [26,27].

#### 5.1.3. Driving Cytokine Storm and Immune Dysregulation

Elevated IL-6 promotes the recruitment and activation of neutrophils, monocytes and lymphocytes into the lungs, amplifying inflammation and causing diffuse lung injury and increased vascular permeability. High IL-6 levels correlate with lymphopenia and impaired adaptive immune responses, contributing to dysregulated immunity and poorer clinical outcomes [28,29].

#### 5.1.4. Crosstalk with Angiotensin and Oxidative Pathways

SARS-CoV-2-mediated downregulation of angiotensin-converting enzyme 2 (ACE2) enhances angiotensin II signaling, which itself induces IL-6 expression via oxidative stress and NF-κB pathways. This interplay between IL-6 and the renin–angiotensin system exacerbates inflammation, edema and endothelial dysfunction in the lungs and other organs [25].

#### 5.1.5. Contribution to Coagulopathy and Multi-Organ Injury

IL-6 influences expression of coagulation factors and adhesion molecules, promoting hypercoagulability and microthrombosis that are common in severe COVID-19. It also stimulates acute phase responses in the liver (e.g., CRP production) and affects metabolic pathways, contributing to systemic inflammation and injury beyond the lungs [30].

#### 5.1.6. Contribution to Lung-Centric Macrophage Activation Syndrome (MAS) in COVID-19 Setting

Unlike classical MAS, which has systemic manifestations characterized by lymphoid organ hyperplasia, hepatosplenomegaly, adenopathy and bone-marrow hemophagocytes, COVID-19-associated MAS appears anatomically compartmentalized to the lungs without the classical organomegaly pattern. IL-6 is produced locally within inflamed lung tissue and bronchoalveolar compartments, contributing to ARDS while biochemically mimicking systemic MAS. This MAS-like hyperinflammation is characterized by hyperferritinaemia, elevated CRP, coagulopathy, and elevated IL-6 [31].

## 6. IL-7 in COVID-19

IL-7, produced by stromal cells, is a key cytokine involved in T-cell development, survival, and homeostasis. It has been explored in cancer immunotherapy to enhance lymphocyte reconstitution, particularly following T-cell–depleting treatments [32]. IL-7 has documented efficacy as an antiviral agent and has shown to restore lymphocyte counts leading to decreased viral load and clinical improvement in several life-threating viral infections [33]. Cytokine profiling studies suggest that IL-7 levels have been elevated in COVID-19, often as a feedback response to T-cell depletion (lymphopenia) [34].

### Therapeutic and Immunological Role of IL-7 in COVID-19

A study showed that IL-7 therapy can increase circulating lymphocyte counts in severe COVID-19 patients with immunosuppression, offering a biological rationale for its use to restore protective immunity [35]. A double-blind, placebo-controlled trial evaluated recombinant human IL-7 (CYT107) in critically ill COVID-19 patients with lymphopenia where they observed that IL-7 was well tolerated and did not precipitate cytokine storm or worsen pulmonary function, highlighting its potential safety profile and effects on lymphocyte recovery [36]. A case report described that IL-7 administration reversed severe lymphopenia and improved T-cell function in a critically ill COVID-19 patient who was identified with a deleterious autosomal dominant mutation in TICAM1, associated with a dysfunctional type I interferon antiviral response [37]. Another study found a positive interaction of IL-2 and IL-7 levels for achieving a satisfactory post-vaccination response in adults with chronic COVID-19 disease [38]. It has also been emphasized that IL-7 has a promising role as a vaccine adjuvant and could potentially enhance the immune responses to vaccines against SARS-CoV-2 [39].

## 7. IL-9 in COVID-19

Although both IL-9 and SARS-CoV-2 are known to cause broncho-alveolar inflammation; there have been variable reports regarding the pathological role of IL-9 in COVID-19. Sadhu S et al. claimed that IL-9 exacerbated airway inflammation caused by SARS-CoV-2 infection in a transgenic forkhead box protein O1 (Foxo1)-deficient mouse model, thus proving the role of a Foxo1-Il-9-mediated Th cell-specific pathway playing a role in COVID-19 [40]. A multiplex cytokine analysis in an Italian cohort found higher IL-9 levels in COVID-19 patients, though the patterns varied [41]. In contrast, another cytokine profiling study did not find any significant difference in serum IL-9 levels between COVID-19 patients and healthy controls [42].

### 7.1. Protective Role of IL-9 in COVID-19

#### Role of IL-9 in the Helminth-Mediated Modulation of COVID-19 Cytokine Storm

A study demonstrated that IL-9 plays a protective, immunoregulatory role in SARS-CoV-2-associated hyperinflammation. Helminth infection induces robust IL-9 production, primarily from Th9 cells, which contributes to the attenuation of the COVID-19-related cytokine storm. IL-9 was shown to suppress excessive pro-inflammatory cytokines such as IL-6, TNF-α, and IL-1β, thereby limiting systemic inflammation and lung immunopathology. Importantly, neutralization of IL-9 abrogated the protective effects of helminth infection, confirming that the anti-inflammatory and disease-ameliorating effects were IL-9-dependent. The findings highlighted IL-9 as a key mediator of helminth-driven immune modulation, capable of restoring immune balance and preventing severe inflammatory responses during SARS-CoV-2 infection [43]. In support of this study, another study by Xiang C et al. proposed that anti-inflammatory molecules from IL-9 dependent Trichinella sprialis (Ts) excretory/secretory (TsES) products could be a novel source for treating such illnesses [44].

## 8. IL-10 in COVID-19

Many studies have claimed IL-10 as one of the most important interleukins for determining the severity and predicting the course of COVID-19 disease, thus serving it as a biomarker for prognosis (along with IL-6) [45,46,47,48]. A study on IL-10 gene polymorphisms stated that specific IL-10 gene variants (e.g., rs1800871, rs1800872, rs1800896) were associated with higher COVID-19 mortality across SARS-CoV-2 variants [49].

### 8.1. Mechanistic and Therapeutic Perspectives of IL-10 in COVID-19

#### 8.1.1. Paradoxical Elevation of Pro-Inflammatory and Immunostimulatory Molecule

Given its well-established anti-inflammatory and immunosuppressive functions [50], the IL-10 showed marked elevation in COVID-19 which can be interpreted as a compensatory response aimed at limiting hyperinflammation and preventing tissue injury. However, the simultaneous increase in IL-10 alongside multiple pro-inflammatory cytokines, together with the association between elevated IL-10 levels and greater disease severity, suggests that IL-10 may be insufficient to effectively restrain inflammation, as reported in other inflammatory conditions [51,52], or may be functioning in a context-dependent manner that departs from its classical anti-inflammatory role [53].

#### 8.1.2. Immunomodulatory Effects of IL-10

IL-10 significantly downregulated IFN-γ, decreased the frequencies of T-cells producing IFN-γ, TNF-α, and IL-2 and down-modulated HLA-DR expression on CD8+ and NK cells in COVID-19 patients, proving its immunomodulating effects [54].

#### 8.1.3. Therapeutic Potential of IL-10

IL-10 has demonstrated anti-fibrotic activity and can mitigate acute lung injury and acute respiratory distress syndrome (ARDS) in COVID-19 patients, owing to which it has been proposed as a potential therapeutic agent for COVID-19 [55].

## 9. IL-17 in COVID-19

IL-17, primarily produced by Th17 as well as γδ T cells and innate lymphoid cells, plays an important role in adaptive immunity and inflammatory responses in the body during infection [56]. Sharif-Askari FS et al. ascertained that high IL-17 level in saliva was associated with COVID-19 severity, need for mechanical ventilation and mortality, thus making IL-17 a non-invasive biomarker of severe COVID-19 [57].

### 9.1. Mechanisms of IL-17 in COVID-19 Pathogenesis

#### 9.1.1. Neutrophil Recruitment and Lung Injury

IL-17 stimulates the production of neutrophil-attracting chemokines such as CXCL1 and CXCL8 (IL-8), leading to excessive neutrophil infiltration into the lungs. Activated neutrophils release reactive oxygen species, proteases, and neutrophil extracellular traps (NETs), thereby exacerbating acute lung injury and ARDS [58,59]. Moreover, Th17 inflammatory response plays an important role in the pathogenesis of COVID-19 pneumonia leading to the release of IL-17 which further promotes pulmonary inflammation by neutrophil and monocyte migration to the lungs, and by activating other cytokine cascades (G-CSF, TNFα, IL-1β and IL-6) [60].

#### 9.1.2. Potential Role in Pulmonary Fibrosis and Post-COVID Sequelae

Persistent IL-17 signaling has been implicated in fibroblast activation and extracellular matrix deposition, suggesting a role in the development of pulmonary fibrosis and long-term lung impairment following severe COVID-19 [61].

## 10. IL-18 in COVID-19

IL-18 has a fundamental role in the clearance of viruses, by promoting IFN-γ production and activation of NK cells, T helper (Th)-1, Th-2, Th-17 cells and regulatory T (T-reg) cells [62]. In addition to adaptive immune responses, IL-18 (along with IL-2) also amplifies innate immune response by enhancing the production of Th-2 cytokines like IL-3, IL-4, IL-9, and IL-13 from NK cells and Th-1 cells [63,64]. Various studies have shown that increased IL-18 has been associated with more severe COVID-19 disease [65,66,67].

### 10.1. Mechanisms of IL-18 in COVID-19 Pathogenesis

#### 10.1.1. NLRP3 Inflammasome Activation

SARS-CoV-2 infection activates the NLRP3 inflammasome in monocytes, macrophages, and epithelial cells, leading to caspase-1-dependent cleavage of pro-IL-18 into its biologically active form, thus resulting in elevated circulating IL-18 in COVID-19 patients and depicting angiotensin-converting enzyme 2 (ACE2) as the principal trigger of the NLRP3 activation [68,69,70,71].

#### 10.1.2. SARS-CoV-2 Spike Protein Induces IL-18-Mediated Cardiopulmonary Inflammation

A study demonstrated that SARS-CoV-2 spike protein induces IL-18 expression via NLRP3 inflammasome activation, which contributes to cardiopulmonary inflammation and cardiac fibrosis in a mouse model. IL-18 inhibition reduced inflammation and fibrosis, suggesting the role of IL-18 in COVID-19 pathogenesis and potential as a therapeutic target [72].

#### 10.1.3. Regulation by IL-18 Binding Protein (IL-18BP)

IL-18 activity is physiologically regulated by IL-18BP, a natural inhibitor. In severe COVID-19, although IL-18BP levels increase, they may be insufficient to neutralize excess IL-18, resulting in sustained inflammatory signaling [66].

#### 10.1.4. Role in Mucosal Immune Response Against SARS-CoV-2

IL-18 levels were inversely correlated with anti-SARS-CoV-2 IgA and IgG detected in nasal fluids of both pediatric and adult patients, suggesting a role for IL-18 in modulating the mucosal immune response against SARS-CoV-2 [73].

#### 10.1.5. Role in SARS-CoV-2 Elicited Intestinal Infection

IL-18 acts as a “double-edged sword” in inflammatory bowel disease, maintaining intestinal homeostasis at baseline but causing severe inflammation when overproduced [74]. The study by Tao W et al. supports the concept that SARS-CoV-2-associated inflammation activates the gut inflammasome–IL-18 axis, contributing to alterations in intestinal microbiota composition [75].

#### 10.1.6. Contribution to Ongoing Immune Dysregulation During Convalescence Period After COVID-19 Infection

A study described the way in which IL-18 was not only associated with acute immune responses but also appeared to reflect a prolonged inflammatory state in recovered COVID-19 donors, supporting its role as a biomarker of sustained immune activation post-infection, because convalescent individuals exhibited a sustained increase in IL-18 levels despite clinical recovery [76].

## 11. IL-27 in COVID-19

IL-27 exerts both pro-inflammatory and protective effects by promoting Th1-mediated immune responses, while simultaneously inducing regulatory functions through upregulation of co-inhibitory receptors on T cells [77]. IL-27 drives interferon-independent, STAT1-dependent inflammatory and antiviral responses in PBMCs and monocytes from patients with severe COVID-19 disease [78]. A case–control study observed that single nucleotide polymorphism of IL-27P28 rs153109 and AA genotypes of IL27 is associated with the susceptibility of SARS-CoV-2 infection but not the severity [79]. Studies primarily show that IL-27, along with IL-6, IL-10, and IL-18, is consistently elevated in COVID-19 patients, but IL-27 levels may not reliably reflect disease severity [80].

### 11.1. Mechanisms of IL-27 in COVID-19 Pathogenesis

#### 11.1.1. Exhibiting Both Inflammatory and Protective Roles in COVID-19

##### o Pro-Inflammatory/Disease-Promoting Effects of IL-27 in COVID-19

IL-27 promotes Th1 polarization and enhances IFN-γ production, contributing to heightened inflammatory responses in COVID-19. It supports the activation and cytotoxic function of CD8^+^ T cells, which, while antiviral, may exacerbate tissue inflammation when excessively activated. Elevated IL-27 levels are associated with immune activation and disease severity, suggesting a role in sustaining inflammation. Through STAT1-dependent signaling, IL-27 can amplify pro-inflammatory gene expression in immune cells, potentially contributing to immunopathology [80].

##### o Anti-Inflammatory/Protective Effects of IL-27 in COVID-19

IL-27 exerts immunoregulatory functions by promoting the differentiation and activation of regulatory T cells (Tregs), thereby limiting excessive immune activation. It induces the expression of co-inhibitory receptors on T cells, contributing to immune checkpoint-mediated regulation and prevention of uncontrolled inflammation. IL-27 influences antibody class switching, supporting effective humoral immunity and viral clearance. By balancing effector and regulatory immune responses, IL-27 contributes to controlled antiviral immunity and may help prevent immune-mediated tissue damage [80].

#### 11.1.2. Role in Multisystem Inflammatory Syndrome in Children (MIS-C)

MIS-C is a rare but life-threatening post-infectious complication of COVID-19 typically manifesting 2–6 weeks after SARS-CoV-2 infection and characterized by persistent fever, laboratory evidence of inflammation, and multi-organ (≥2) involvement in individuals < 21 years old. MIS-C shares features like rash and strawberry tongue with Kawasaki Disease but typically affects older children and presents more frequently with shock and gastrointestinal distress [81]. A study focused on IL27 gene expression stated that IL-27 is a potent antiviral and anti-inflammatory cytokine that differentiates MIS-C from other febrile illnesses, highlighting the broader relevance of IL-27 in SARS-CoV-2-related immune dysregulation [82].

## 12. IL-33 in COVID-19

IL-33, an alarmin localized within the lung epithelium, plays a critical role in limiting post-injury inflammation by mediating Foxp3^+^ regulatory T-cell-dependent control of local cytokine production and myeloid cell responses [83]. After being released from alveolar type II epithelial cells and alveolar macrophages, IL-33 plays a critical role in respiratory inflammation and tissue injury by activation of mast cells, Th2 cells, M2 macrophages, eosinophils, leading to increased production of IL-4, IL-9, IL-10, IL-13, and TGF-β [84]. Additionally, it has been demonstrated that severe trauma triggers a rapid surge of IL-33, which activates type 2 innate lymphoid cells (IL-C2s) in the lungs to produce IL-5, thus generating IL-33–IL-C2–IL-5–neutrophil axis which drives early post-trauma lung inflammation and damage [84]. Studies have also shown that elevated IL-33 is a significant predictive marker of adverse outcome in COVID-19 patients [85]. In another study, IL-33 was shown to be associated with COVID-19 severity in African American males coupled with higher concentrations of renal toxicity markers like glutathione S-transferase (GST) and osteopontin, thus establishing its putative interference in multi-systemic damages of SARS-CoV-2 infection [86]. Effects of IL-33 in COVID-19 involve neutrophil infiltration, dendritic cell activation, Th17 differentiation, and inflammatory cell recruitment. IL-33/ST2 signaling may induce inflammatory cascades and complications such as vasoembolic conditions [87]. Another study reported that SARS-CoV-2 spike peptide mixture could induce IL-33 secretion in the culture supernatant of PBMCs from seropositive individuals, suggesting that immune cells may also be a source of IL-33 in COVID-19 [88]. In a recent study, IL-33 has been identified as a critical driver that synergizes with IL-3 to significantly upregulate CD25 (IL-2Rα) expression on human basophils and hence serve as a marker of severe COVID-19, highlighting an emerging immunopathogenic role of IL-33 in innate effector cells [89].

## 13. IL-36 in COVID-19

IL-36, a member of the IL-1 cytokine family, acts as a pro-inflammatory mediator that may contribute to hyperinflammation and tissue injury in COVID-19 [90].

### 13.1. Mechanisms of IL-36 in COVID-19 Pathogenesis

#### 13.1.1. Contribution in Intestinal and Cutaneous Manifestations of COVID-19

IL-36 primarily contributes in ACE2 regulation and intestinal inflammation in COVID-19 patients [90]. Additionally, the presence of ACE2 and SARS-CoV-2 RNA in blood vessels [91,92], together with the expression of IL-36γ and IL-36R in human dermal microvascular endothelial cells [93,94], suggests that endothelial SARS-CoV-2 infection may trigger IL-36-mediated leukocyte recruitment and cutaneous manifestations in COVID-19.

#### 13.1.2. Positive Pro-Inflammatory Feedback Loops and Inflammation Amplification

IL-36 promotes IL-6 and IL-8 production, and is itself induced by IL-1β and IL-6, forming a pro-inflammatory feedback loop that can sustain and escalate inflammatory responses in severe COVID-19 disease. IL-36 can promote differentiation and activity of Th1 and Th17 cells, potentially exacerbating inflammatory responses [90].

## 14. Role of Interleukins in SARS-CoV-2 Variants

The WHO, in collaboration with the SARS-CoV-2 Interagency Group (SIG) and the Centers for Disease Control and Prevention (CDC), devised a classification system for SARS-CoV-2 variants categorizing them as “Variants of Interest (VOI),” “Variants of Concern (VOC),” “Variants of High Consequence (VOHC),” and “Variants Being Monitored (VBM)” [95]. ILs were extensively studied in the context of SARS-CoV-2 VOCs because these variants significantly altered cytokine profiles, directly influencing disease severity and transmission. VOCs exhibited heightened transmissibility, exacerbated illness, significantly reduced neutralization by antibodies and a lowered efficacy of treatments and/or vaccines [96]. The Alpha variant (B.1.1.7) was the first variant of concern and was identified in the United Kingdom in late December 2020. The Beta variant (B.1.351) was subsequently reported in South Africa in December 2020, followed by the emergence of the Delta variant (B.1.617.2) in India during the same period. The Omicron variant (B.1.1.529) was later identified in South Africa in November 2021 [97].

It has been demonstrated in studies that IL-1α, IL-4, IL-9 and IL-16 were significantly elevated in Delta versus the Omicron variant, with full vaccination significantly decreasing levels of IL-7 and IL-8 in the Omicron variant [98]. Cytokine profiling showed that IL-6, IL-10, and IL-18 levels remained persistently elevated across Delta and Omicron infections, making them reliable markers of SARS-CoV-2-induced inflammation in multiple variant waves [34].

SARS-CoV-2 variants, particularly Delta and Omicron, elicit a host transcriptional response that is distinct from the ancestral (wild-type) strain, characterized by heightened inflammatory gene expression especially those encoding pro-inflammatory interleukins (e.g., IL-6, IL-1β, IL-8), chemokines (CXCL8, CCL2), and NF-κB-regulated mediators. This enhanced inflammatory signature suggests that variant-specific mutations alter host–pathogen interactions, leading to exaggerated cytokine and chemokine signaling. Such changes are consistent with clinical observations of pronounced systemic inflammation and immune dysregulation in variant-driven COVID-19 [99].

Another study comparing the wild-type SARS-CoV-2 spike protein (WT-S) and B.1.1.529 Omicron (Omicron-S) demonstrated that WT-S induces higher IL-6 production in macrophages compared with Omicron spike protein, suggesting that mutations of spike protein associated with wild type and SARS CoV-2 variants have a great impact on the pro-inflammatory ability of the virus [100].

Another cytokine profiling study showed that IL-18, IL-7, IL-8, IL-1β were significantly lower in Omicron infections vs. Wuhan strain, thus aligning with milder disease outcomes seen clinically in this variant. Delta infections were associated with more lymphopenia and reduced IL-7-mediated homeostatic signals relative to Omicron, consistent with greater impairment of adaptive immune responses [101]. In the Wuhan and Delta cohorts, IL-6 showed correlations with levels of Galectin-3 (Gal-3), a proinflammatory mediator, indicating that IL-6 remains a central cytokine linked with heightened inflammation in these variants [34,101]. It has also been concluded that serum IL-6 may serve as a recovery indicator in COVID-19 patients infected with Omicron XBB sub-variants as elevated IL-6 correlated with recovery patterns [102].

Omicron BA.1 and BA.2 sub-variants showed broad interleukin activation profile primarily involving elevated levels of IL-2, IL-4 and IL-10, suggesting that host interleukin signaling remains engaged despite extensive spike mutations, and that adaptive immune signaling involving ILs contributed to T-cell proliferation and humoral immunity during infection with these subvariants [103]. Clinical data show that IL-2, IL-4, IL-6, and IL-10 levels were generally higher in patients infected with earlier Omicron subvariants BA.1 and BA.2 than those with BA.4/BA.5, indicating that more recent Omicron lineages may trigger less pronounced IL-driven inflammation in some cohorts, thus reflecting both viral immune evasion adaptations and the effects of widespread host immunity, including vaccination and prior infection [103,104]. While not subvariant-specific, IL-6 continues to serve as a core pro-inflammatory interleukin in Omicron infections and is relevant to evaluating host inflammatory status in BA.1, BA.2, BA.4, and BA.5 contexts [14]. IL-10 gene polymorphisms affect COVID-19 outcomes differently across variants with certain IL-10 promoter variants being associated with higher mortality risk in Omicron BA.4 infections, underscoring the role of regulatory ILs in modulating disease severity for specific Omicron lineages [49].

A study associating gene polymorphisms with mortality showed that IL-10 polymorphisms significantly influenced severity and mortality in COVID-19, but that the strength and direction of association varied by SARS-CoV-2 variant (Alpha, Delta, Omicron BA.5). IL-10 rs1800896 was linked to worse outcomes mainly in Delta and Omicron BA.5, not in Alpha, whereas IL-10 rs1800871 was associated with increased mortality in Alpha and delta variants, but not in the Omicron BA.5 variant [49].

A study from China also demonstrated that higher levels of IL-6 and IL-10 are associated with increased mortality and remain persistently high over the course of hospitalization in Omicron-associated COVID-19 cases. Negative correlations were observed between specific antibody levels and IL-1β and IL-10, suggesting that greater inflammatory cytokine expression may coincide with impaired humoral immune responses. IL-2R and IL-10 levels were inversely correlated with T-lymphocyte subset counts (CD3^+^, CD4^+^, CD8^+^), linking these cytokines to lymphocyte depletion in severe disease [105].

It has also been demonstrated that, although absolute IL-6 values were highest for the Omicron variant, followed by the Delta, Beta and Alpha variants (lowest), the proportion of patients with elevated IL-6 decreased from Alpha to Omicron, suggesting a shift toward milder systemic inflammatory responses over time. This pattern suggests a trend toward reduced severity even as IL-6 remains a marker of inflammation [106].

An interesting finding that was seen during the evolution of the Omicron variant was ‘Omicron breakthrough mild infection in vaccinated individuals’. A simple explanation for this finding stems from the fact that follicular helper T (Tfh) cells play a central role in inducing high-affinity neutralizing antibodies against SARS-CoV-2 virus [107]. It has been observed that early viral replication during breakthrough infection triggers an IL-6-driven inflammatory response and, paradoxically, although IL-6 is a pro-inflammatory cytokine typically linked with disease severity, in this situation IL-6 promotes the expansion of circulating Tfh cells and rapidly reactivates memory B cells, thereby leading to accelerated production of high-affinity neutralizing antibodies, limiting disease severity and promoting faster viral clearance. Thus, IL-6 in this setting reflects effective immune recall and antibody maturation, explaining why infection can occur despite vaccination, yet remain clinically mild with strong neutralizing activity [108].

Table 1 summarizes the roles of interleukins in different SARS-CoV-2 variants [34,49,98,99,101,102,105,106,108] and Table 2 demonstrates the roles of combination interleukins in COVID-19 and SARS-CoV-2 variants [14,34,98,103,109,110]. 

In summary, elevated levels of IL-1α, IL-1β, IL-4, IL-8, IL-9, IL-16, IL-18 have been documented in the Delta variant as compared with the Omicron variant. Several studies suggest that IL-6 demonstrated constant elevation across all SARS-CoV-2 variants compared when with the ancestral Wuhan strain, although the magnitude of this increase may vary across populations and clinical contexts. IL-10 showed consistent elevation in COVID-19 patients regardless of viral genetic differences. Among the Omicron variant, levels of IL-1β and IL-6 have been positively associated with disease severity.

## 15. Role of Anti-Interleukins in COVID-19 Treatment

### 15.1. IL-1 Blockers

#### 15.1.1. Anakinra (Recombinant IL-1 Receptor Antagonist)

Anakinra blocks the biologic activity of IL-1α and IL-1β by competitively inhibiting their binding to the interleukin-1 type I receptor [111]. A Cochrane review analyzed four randomized controlled trials (RCTs) of anakinra and found little or no increase in the proportion of patients showing clinical improvement by day 28 when compared with standard care or placebo. In one RCT, anakinra showed a possible reduction in severe progression of disease by ≥60 days, but this is of low certainty and limited to a single study. It has an acceptable safety profile but pooled estimates did not show a clear mortality reduction by day 28 or at later follow-ups, leading to an uncertain overall effect on all-cause mortality [112].

#### 15.1.2. Canakinumab

Canakinumab is a recombinant human monoclonal antibody that specifically targets and neutralizes IL-1β, a key pro-inflammatory cytokine, thereby preventing it from interacting with its receptor and leading to suppression of IL-1β-mediated inflammation. A Cochrane review found that the effect of Canakinumab after day 28 on clinical improvement and all-cause mortality was uncertain [112]. Another observational cohort concluded that canakinumab is associated with improved lung oxygenation, reduced inflammation and faster hospital discharge in COVID-19 pneumonia patients. However, because there is no randomized control group, the study could not definitively prove efficacy and improvements might also reflect natural recovery or effects of other treatments received by patients [113].

### 15.2. IL-6 Inhibitors

#### 15.2.1. Tocilizumab

Tocilizumab is a recombinant humanized monoclonal antibody that binds to both soluble and membrane-bound IL-6 receptors, thereby inhibiting IL-6-mediated signaling pathways involved in inflammation and immune activation [114]. Tocilizumab has been used to treat severe or critical COVID-19 cases, particularly where elevated IL-6 may drive an inflammatory cytokine storm [115]. A systematic review found that tocilizumab reduced the all-cause mortality at around 28 days in hospitalized COVID-19 patients when compared with standard care or placebo, with reassuring safety signals, although there was not much benefit in clinical improvement by day 28 [116]. Another systematic review and meta-analysis also claimed that tocilizumab-dominated IL-6 blockade is associated with lower death rates, particularly in severe (but not critical) COVID-19 cases [117]. The WHO recommends tocilizumab only for severe or critical COVID-19 alongside standard care (oxygen, corticosteroids) based on its high-certainty evidence of reduced mortality [118].

#### 15.2.2. Sirukumab

Sirukumab is a human monoclonal antibody that binds and sequesters free IL-6, preventing association with the IL-6 receptor that triggers downstream inflammatory pathways, thus reducing harmful inflammation in hospitalized COVID-16 patients. A phase 2, randomized, double-blind, placebo-controlled clinical trial evaluated sirukumab in critical COVID-19 cases and found no statistically significant difference in time to sustained clinical improvement versus placebo, thus proving that COVID-19 cannot be treated by simply neutralizing circulating IL-6 alone [119]. Another exploratory biomarker study from a sirukumab trial observed that critical COVID-19 cases without detectable sirukumab-induced IL-4 levels are associated with fewer inflammatory chemokines, potentially aligning with better outcomes with sirukumab [120].

### 15.3. Therapeutic Potential of IL-22 in COVID-19

IL-22 is a cytokine in the IL-10 family that primarily acts on non-immune cells such as epithelial and fibroblast cells in the lungs via the IL-22R1/IL-10R2 receptor complex, leading to anti-apoptotic, regenerative and barrier-protective effects in tissues. Studies have described how IL-22 can reduce COVID-19 pneumonia severity through immunomodulation, anti-inflammation and tissue regenerative and repair functions, but the mechanistic details and clinical trial evidence specific to SARS-CoV-2 are limited and much of the support remains theoretical or preclinical [121].

Studies demonstrate that the numbers of IL-22R1^+^ myeloid dendritic cell subsets (mDC1, mDC2), plasmacytoid dendritic cells (pDCs), and IL-22R1^+^ intermediate, classical, and non-classical monocytes are significantly elevated in COVID-19 patients compared with healthy controls at disease presentation, highlighting an active IL-22-responsive immune landscape. Notably, increased proportions of mDC2 cells and IL-22R1^+^ non-classical monocytes exhibit elevated HLA-DR expression, indicating enhanced immune activation and antigen-presenting capacity [122]. Additionally, in patients with non-severe SARS-CoV-2 infection, the number of IL-22R1^+^ intermediate monocytes has been found to be negatively correlated with inflammatory markers, including IFN-α, C-reactive protein (CRP), and IL-6, supporting a protective and inflammation-limiting role of IL-22 signaling [121]. Beyond immune modulation, IL-22 directly affects viral susceptibility at the tissue level. Experimental evidence demonstrates that IL-22 signaling through the IL-22/IL-22R1 pathway reduces the expression of key SARS-CoV-2 entry receptors, including ACE2 and TMPRSS2, while simultaneously enhancing the expression of antiviral proteins in target cells. This mechanism suggests that IL-22 not only modulates immune responses but also limits viral entry and replication, thereby contributing to host defense [123].

Recent studies have highlighted the important involvement of innate lymphoid cells (ILCs) in the immunopathology of COVID-19. Analysis of peripheral blood from COVID-19 patients indicates that individuals with severe disease exhibit significantly reduced numbers of ILC precursor cells and mature ILCs compared with patients experiencing mild infection. Notably, ILCs from severely infected patients display elevated expression of CD69, a marker associated with cell activation and tissue homing. The observed increase in CD69^+^ ILCs, together with a reduction in circulating ILCs, suggests enhanced migration and activation of ILCs within lung tissues during severe SARS-CoV-2 infection [124]. ILCs are recognized as a major source of IL-22 during pulmonary infections, and as SARS-CoV-2 primarily targets the respiratory epithelium, it has been speculated that the migration of IL-22-producing ILCs from the circulation to the lungs may represent a protective response aimed at limiting epithelial damage and supporting tissue regeneration during infection [125]. However, current evidence regarding the role of IL-22 and ILCs in COVID-19 is largely derived from peripheral blood analyses, which may not fully reflect immune dynamics within lung tissues. Therefore, further studies focusing on local tissue-level responses are urgently needed to elucidate the precise contribution of ILC-derived IL-22 to the pathogenesis and potential treatment of SARS-CoV-2 infection.

Experiments in lung injury models (e.g., influenza infection) demonstrate that IL-22 induces anti-apoptotic proteins and growth-associated genes that help epithelial layers recover faster; hence the lack of IL-22 leads to worsened epithelial damage and delayed repair, suggesting a similar role in COVID-19 [126].

### 15.4. Therapeutic Potential of IL-17 in COVID-19

Acute lung injury in patients with severe COVID-19 is characterized by intense inflammation and extensive tissue damage within the respiratory tract. This pathological process has been strongly associated with T-helper 17 (Th17) cell-mediated immune responses, primarily through the actions of IL-17. Differentiation of Th17 cells during the early stages of inflammation is largely mediated by IL-6-induced activation of the JAK2/STAT3 signaling pathway, which enhances the transcription of IL-17A, IL-17F, and IL-22. Downstream signaling initiated by IL-17A stimulates the production of a wide array of pro-inflammatory cytokines, chemokines, growth factors, and antimicrobial peptides from multiple cell types which include CXCL1, CXCL5, CXCL12, MIP-3α, G-CSF, GM-CSF, IL-1, IL-6, IL-8, TNF-α, and IP-10, that collectively promote granulopoiesis, immune-cell recruitment, microbial clearance, mucosal defense, and tissue inflammation [127,128]. In COVID-19, persistent IL-17 signaling contributes to a hyperinflammatory state, exacerbating lung injury, and promoting disease severity in COVID-19 patients. Therefore, IL-17 inhibition represents a potential immunomodulatory strategy to reduce lung pathology and improve clinical outcomes in patients with severe COVID-19 [129].

The BISHOP phase-II controlled trial was conducted to evaluate secukinumab, an anti-IL17A monoclonal antibody, for the treatment of severe COVID-19 and it was observed that, although secukinumab decreased pulmonary embolism in male patients, it did not show clear efficacy in improving key clinical outcomes in this trial [130]. A pilot study undertaken on the IL-17 inhibitor netakimab showed that although it was associated with improved oxygenation and reduction in inflammatory markers without notable adverse events in severe COVID-19, it did not affect the need for mechanical ventilation and mortality [131].

A clinical trial has been evaluating baricitinib, a JAK1/JAK2 inhibitor, which has been identified through an artificial intelligence-based screening approach due to its ability to inhibit viral entry into alveolar epithelial cells and its potential to suppress excessive inflammatory responses in patients [132]. However, baricitinib has been associated with an increased risk of infections, malignancies, and thrombotic events, primarily reported during chronic drug administration. To date, the safety of the acute administration of these agents in critically ill patients has not been fully established, necessitating cautious consideration if they are used to treat severe COVID-19. Furthermore, the timing of intervention is likely to be critical; early administration may offer greater benefit by preventing progression to severe or fatal disease, provided that treatment is discontinued before the risk of opportunistic infections becomes substantial [133].

## 16. Cellular Immune Responses After COVID-19 Vaccination

A longitudinal study in Japan evaluated cellular immune responses to severe SARS-CoV-2 spike protein after COVID-19 mRNA primary and booster vaccination. It was observed that, although long-term care facility (LTCF) residents exhibited significantly lower IL-2 and IL-6 levels than healthcare workers after the primary vaccination, booster vaccination increased IL-2 and IL-6 levels in LTCF residents comparable to those in healthcare workers. Additionally, IL-2 levels correlated positively with spike-specific antibody (IgG) levels and neutralizing activity for wild-type and Delta SARS-CoV-2. IL-6 increased after primary vaccination showing vaccine-induced innate/Th1 cell engagement but, after booster, IL-6 did not significantly increase further in LTCF residents and outpatients, and declined in healthcare workers compared with pre-booster levels. IL-6 levels were positively correlated with IgG and neutralizing antibody activity after primary vaccination. However, neutralizing activity against the Omicron variant showed a negative correlation with IL-6. IL-10 levels were not significantly different from the initial values after primary vaccination but demonstrated a significant increase after booster vaccination. IL-10 levels did not correlate with IgG levels or neutralizing antibody activity, unlike the pro-inflammatory interleukins. Age negatively correlated with pro-inflammatory cytokines IL-2 and IL-6 after both primary and booster vaccination, indicating that immunosenescence affects vaccine responses [134].

Another study also revealed enhanced IL-2 expression from 4 to 48 h alongside T-cell responses following mRNA vaccines, indicating its involvement in a Th1-skewed cellular immune profile that assists viral clearance and cooperation with cytotoxic T cells [135]. Certain studies have found a transient rise in IL-15 soon after mRNA 1st vaccination, correlating with improved cellular and humoral immunity in response to vaccination. Transient increases in IL-6 were observed after the 2nd vaccination, which can be considered as part of the initial innate response that bridges toward adaptive T-cell activity, although persistent high IL-6 is more associated with pathogenic inflammation than protective immunity [136]. Studies have also observed that T-cell IL-21 response was positively associated with SARS-CoV-2-specific B cell response and spike S1-specific IgG antibody levels, demonstrating robust B cell-mediated immune responses in chronic kidney disease (CKD) patients and kidney transplant recipients [137]. Studies also claim that the interaction of IL-2 and IL-7 is important for achieving post-vaccination immunity, especially in adults with chronic diseases and that higher IL-7 levels post-vaccination may boost the longevity of vaccine-induced cellular immunity, particularly in adults with otherwise suboptimal immune profiles [38].

In summary, the above studies have demonstrated that elevated IL-15 levels were observed after the first dose of mRNA vaccine, with booster doses showing relative increases4 in IL-2 and IL-6 levels but a significant increase in IL-10 levels. Only IL-2, IL-6, IL-15 and IL-21 levels were positively correlated with IgG and neutralizing antibody activity after vaccination, but further studies need to be undertaken in order to establish their association with a significant protective antibody response to vaccination. Longevity of post vaccination immunity has been determined by the combination of IL-2 and IL-7.

## 17. Conclusions

Interleukins play a central role in the immunopathogenesis of COVID-19 and in shaping host responses to different SARS-CoV-2 variants. Patients with SARS-CoV-2 infection exhibit markedly elevated levels of multiple pro- and anti-inflammatory interleukins, reflecting immune activation that can serve both as a biomarker of disease severity and progression and as a potential therapeutic target. Dysregulated interleukin signaling, particularly involving IL-6, IL-1β, IL-17, and IL-10, contributes to hyperinflammation, immune imbalance, and the development of systemic complications, including ARDS, secondary infections, and multi-organ dysfunction. Emerging evidence indicates that distinct SARS-CoV-2 variants induce variant-specific cytokine and interleukin profiles, which although to some extent explain differences in transmissibility, clinical severity, and immune evasion; but it still remains unclear to what extent these patterns reflect intrinsic variant biology versus confounding influences such as vaccination status, prior exposure, and evolving standards of care. Similarly, although combined interleukin signatures have been associated with hyperinflammatory responses and increasing clinical severity in COVID-19, and predominantly the Delta SARS-CoV-2 variant, it is still difficult to state whether combined ILs versus a single IL can significantly alter the prognosis and increase mortality rates, as the strength of inference of statistical models in the published data is not satisfactory.

While excessive pro-inflammatory interleukin responses are associated with severe disease, inadequate or delayed regulatory cytokine responses can further exacerbate immune-mediated tissue damage. Thus, the balance between inflammatory and anti-inflammatory interleukins is critical in determining clinical outcomes.

A deeper understanding of interleukin-mediated immune pathways in COVID-19 is essential for identifying reliable prognostic markers and for guiding targeted immunomodulatory therapies. Although cytokine-directed treatments have shown promise, further research is required to refine patient selection, optimize timing, and tailor interventions according to disease stage and viral variant. Continued efforts in this area will be crucial for developing effective strategies to control inflammation, reduce complications, and improve clinical outcomes in COVID-19 and future SARS-CoV-2 variant driven infections.

## Figures and Tables

**Table 1:** Roles of interleukins in different SARS-CoV-2 variants.

Interleukin	SARS-CoV-2 Variant(s)	Observed Role/Findings (Contextualized)	Population/Sample Type	Timing of Measurement	Vaccination Status	Study Design	Reference No(s).
IL-1α	Delta vs. Omicron	Higher circulating IL-1α concentrations reported in Delta infections compared with Omicron, consistent with stronger innate inflammatory activation; In the Omicron group, IL-1α (along with other cytokines) increased with disease severity	80 Hospitalized COVID-19 patients; Two variant groups: Delta (n = 50) and Omicron (n = 30); plasma samples	Acute phase	✓ Omicron group: vaccinated vs. unvaccinated comparison was performed. ✓ Delta group: vaccination details not reported	Retrospective observational study	[98]
IL-1β	Delta, Omicron, Alpha	IL-1β–associated inflammatory signaling was upregulated in infections caused by SARS-CoV-2 variants compared with wild-type strains, particularly in symptomatic patients	51 COVID-19 cases included in the transcriptome analysis; Whole blood-Whole blood RNA was analyzed using microarray platforms	Acute phase	Majority unvaccinated: authors explicitly excluded vaccinated individuals in certain subset comparisons (e.g., asymptomatic VOC vs. wild type).	Comparative observational transcriptomic study	[99]
IL-1β	Delta, Omicron, Wuhan strain	Delta variant infection induced stronger IL-1β expression; Omicron variant showed a relatively attenuated IL-1β response	SARS-CoV-2 clinical isolates from infected human cases; Replication-competent SARS-CoV-2 isolates	Acute phase	Not applicable/not addressed directly.	Comparative experimental laboratory study	[101]
IL-1β	Omicron	IL-1β levels were positively associated with disease severity; higher IL-1β occurred in patients with weaker humoral responses; Elevated IL-1β alongside other cytokines reflects an exuberant inflammatory response in more severe Omicron infection	140 hospitalized adult patients with confirmed Omicron variant SARS-CoV-2 infection; serum samples	Acute phase; first Omicron wave in China	74% (104/140) of patients had received SARS-CoV-2 vaccination	Observational, cross-sectional with longitudinal follow-up	[105]
IL-2R	Omicron	Higher soluble IL-2R levels associated with lower CD3^+^, CD4^+^ and CD8^+^ T-cell counts, suggesting lymphocyte dysregulation in severe Omicron infection	140 hospitalized adult patients with confirmed Omicron variant SARS-CoV-2 infection; serum samples	Acute phase; first Omicron wave in China	74% (104/140) of patients had received SARS-CoV-2 vaccination	Observational, cross-sectional with longitudinal follow-up	[105]
IL-4	Delta vs. Omicron	Higher IL-4 concentrations reported in Delta infections compared with Omicron, indicating a stronger Th2-skewed response	80 Hospitalized COVID-19 patients; Two variant groups: Delta (n = 50) and Omicron (n = 30); plasma samples	Acute phase	✓ Omicron group: vaccinated vs. unvaccinated comparison was performed. ✓ Delta group: vaccination details not reported	Retrospective observational study	[98]
IL-6	Delta, Omicron, Alpha Ancestral Wuhan Strain	Consistent elevation across all variants; Highest in severe disease and particularly pronounced in the Alpha variant; Considered as “constant marker” of COVID-19 immune activation	340 hospitalized COVID-19 patients and 51 healthy donors; Plasma and nasopharyngeal swabs	Acute phase	All COVID-19 patients were unvaccinated	Retrospective observational study	[34]
IL-6	Delta, Omicron, Alpha	All SARS-CoV-2 variants induced higher IL-6 gene expression relative to the ancestral Wuhan strain.	51 COVID-19 cases included in the transcriptome analysis; Whole blood-Whole blood RNA was analyzed using microarray platforms	Acute phase	Majority unvaccinated: authors explicitly excluded vaccinated individuals in certain subset comparisons (e.g., asymptomatic VOC vs. wild type).	Comparative observational transcriptomic study	[99]
IL-6	Omicron	Elevated IL-6 levels correlated with: increased disease severity, higher inflammatory burden, worse clinical outcomes.	450 Asian COVID-19 patients infected with SARS-CoV-2 Omicron XBB sub-variants; peripheral blood samples	Acute phase	Study reflected the post-vaccination Omicron era; Vaccination status was reported but not deeply stratified	Retrospective observational cohort study	[102]
IL-6	Omicron	IL-6 levels were significantly elevated in critical patients compared with severe cases;	140 hospitalized adult patients with confirmed Omicron variant SARS-CoV-2 infection; serum samples	Acute phase; first Omicron wave in China	74% (104/140) of patients had received SARS-CoV-2 vaccination	Observational, cross-sectional with longitudinal follow-up	[105]
IL-6	Alpha, Beta, Delta, Omicron	Decreasing proportion of patients with high IL-6 values from Alpha through Omicron; mean IL-6 levels for Omicron were elevated in a small patient subgroup reflecting rare severe disease.	40,133 adults with confirmed SARS-CoV-2 infection; serum samples	Not standardized or fully reported.	Vaccination data were not reported or analyzed	Retrospective observational study	[106]
IL-6	Omicron	Elevated IL-6 levels during acute Omicron pneumonia were positively correlated with increased circulating follicular helper T cell (cTfh) frequency and stronger neutralizing antibody responses	Adult hospitalized but non–critically ill patients with Omicron variant SARS-CoV-2 infection who developed COVID-19 pneumonia despite prior vaccination; Peripheral blood samples	Acute phase	All COVID-19 patients had received at least two doses of SARS-CoV-2 vaccine prior to infection, consistent with breakthrough Omicron infection.	Observational cohort study	[108]
IL-7	Delta vs. Omicron	Higher IL-7 levels in Omicron-infected patients compared with Delta cases.	80 Hospitalized COVID-19 patients; Two variant groups: Delta (n = 50) and Omicron (n = 30); plasma samples	Acute phase	✓ Omicron group: vaccinated vs. unvaccinated comparison was performed. ✓ Delta group: vaccination details not reported	Retrospective observational study	[98]
IL-7	Delta, Omicron, Wuhan strain	Compared with the Wuhan strain and Delta variant, Omicron infection showed relatively preserved or less suppressed IL-7–associated signaling, consistent with a more balanced immune profile.	SARS-CoV-2 clinical isolates from infected human cases; Replication-competent SARS-CoV-2 isolates	Acute phase	Not applicable/not addressed directly.	Comparative experimental laboratory study	[101]
IL-8	Delta vs. Omicron	Significantly elevated IL-8 concentrations in patients infected with the Delta variant relative to those infected with Omicron suggesting more severe pulmonary inflammation and higher rates of hypoxemia in Delta variant	80 Hospitalized COVID-19 patients; Two variant groups: Delta (n = 50) and Omicron (n = 30); plasma samples	Acute phase	✓ Omicron group: vaccinated vs. unvaccinated comparison was performed. ✓ Delta group: vaccination details not reported	Retrospective observational study	[98]
IL-8	Delta, Omicron, Alpha	IL-8 is prominently increased in SARS-CoV-2 variants relative to the wild-type strain; key interleukin responsible for neutrophil chemotaxis and activation	51 COVID-19 cases included in the transcriptome analysis; Whole blood - Whole blood RNA was analyzed using microarray platforms	Acute phase	Majority unvaccinated: authors explicitly excluded vaccinated individuals in certain subset comparisons (e.g., asymptomatic VOC vs. wild type).	Comparative observational transcriptomic study	[99]
IL-8	Delta, Omicron, Wuhan strain	Wuhan and Delta infections tended to elicit higher IL-8 levels than Omicron; IL-8 showed positive correlations with Gal-3 (a soluble pro-inflammatory glycan-binding protein) in the Wuhan cohort, suggesting IL-8 levels tracked with broader inflammatory activity	SARS-CoV-2 clinical isolates from infected human cases; Replication-competent SARS-CoV-2 isolates	Acute phase	Not applicable/not addressed directly.	Comparative experimental laboratory study	[101]
IL-9	Delta vs. Omicron	Higher IL-9 concentrations reported in Delta infections relative to Omicron, aligning with enhanced airway-associated inflammation	80 Hospitalized COVID-19 patients; Two variant groups: Delta (n = 50) and Omicron (n = 30); plasma samples	Acute phase	✓ Omicron group: vaccinated vs. unvaccinated comparison was performed. ✓ Delta group: vaccination details not reported	Retrospective observational study	[98]
IL-10	Delta, Omicron, Alpha Ancestral Wuhan	IL-10 showed consistent elevation in COVID-19 patients across all variants; serve as a “constant marker” of COVID-19 infection, regardless of viral genetic differences.	340 hospitalized COVID-19 patients and 51 healthy donors; Plasma and nasopharyngeal swabs	Acute phase	All COVID-19 patients were unvaccinated	Retrospective observational study	[34]
IL-10	Alpha, Delta, Omicron BA.5 variant	High-producer genotypes of IL-10 were associated with more severe COVID-19, particularly in infections with later variants.	Patients with confirmed SARS-CoV-2 infection from the Middle Eastern region; Peripheral venous blood samples	Acute phase	Vaccination status was not comprehensively stratified or adjusted	Observational, case–control genetic association study.	[49]
IL-10	Omicron	Dual role for IL-10 in Omicron infection: Protective by limiting cytokine-mediated tissue injury. Potentially detrimental when excessively elevated, by suppressing effective antiviral immunity in severe and critical disease.	140 hospitalized adult patients with confirmed Omicron variant SARS-CoV-2 infection; serum samples	Acute phase; first Omicron wave in China	74% (104/140) of patients had received SARS-CoV-2 vaccination	Observational, cross-sectional with longitudinal follow-up	[105]
IL-16	Delta vs. Omicron	Higher IL-16 concentrations reported in Delta compared with Omicron, suggesting stronger T-cell chemoattractant signaling	80 Hospitalized COVID-19 patients; Two variant groups: Delta (n = 50) and Omicron (n = 30); plasma samples	Acute phase	✓ Omicron group: vaccinated vs. unvaccinated comparison was performed. ✓ Delta group: vaccination details not reported	Retrospective observational study	[98]
IL-18	Delta, Omicron, Alpha Ancestral Wuhan	IL-18 is a conserved but variably amplified inflammatory mediator across SARS-CoV-2 variants, supporting its role in COVID-19–associated innate immune dysregulation.	340 hospitalized COVID-19 patients and 51 healthy donors; Plasma and nasopharyngeal swabs	Acute phase	All COVID-19 patients were unvaccinated	Retrospective observational study	[34]
IL-18	Delta, Omicron, Wuhan strain	Patients infected with the Wuhan strain and the Delta variant both exhibited significantly elevated IL-18 levels compared with healthy controls; Although IL-18 was also elevated in Omicron-infected individuals versus healthy controls, the magnitude of increase was lower than that observed in those infected with the Wuhan strain or Delta variant.	SARS-CoV-2 clinical isolates from infected human cases; Replication-competent SARS-CoV-2 isolates	Acute phase	Not applicable/not addressed directly.	Comparative experimental laboratory study	[101]

**Table 2:** Roles of Combination Interleukins in COVID-19 and SARS-CoV-2 variants.

Interleukin Combination	Observed Role in COVID-19 and SARS-CoV-2 Variants	Statistical Approach Used in Cited Study	Reference No(s).
IL-2, IL-4, IL-6, IL-10	Elevated together in COVID-19 patients compared to healthy controls	Systematic review and meta-analysis of observational studies; weighted mean difference (WMD) and corresponding 95% confidence interval (CI) were calculated to compare the difference in serum interleukin levels; Heterogeneity was assessed using the I^2^ statistic; a fixed-effects model was applied when I^2^ < 50%, whereas a random-effects model was used otherwise. No unified cutoff values reported. No predictive Area Under the Curve (AUC) reported.	[14]
IL-6, IL-8, IL-10	Levels of these three interleukins were significantly higher in severe COVID-19 vs. non-severe, correlating with disease severity and potentially poor outcomes.		[14]
IL-1β, IL-6, IL-8	Elevated together in nonsurvivor COVID-19 patients, suggesting a combined interleukin signature predicts poor prognosis.		[14]
IL-1β + IL-6	Both interleukins cooperate in driving the COVID-19 cytokine storm i.e., IL-1 enhances IL-6 and downstream inflammation, contributing to systemic hyperinflammation.	Observational, cross-sectional comparative design; Logistic regression model used to estimate associations between cytokine levels and severe/critical disease.; AUC values were higher for IL-6 than IL-1 indicating superior predictive utility.; ROC-derived cut-off points reported for IL-6 were selected using Youden’s index to optimize sensitivity and specificity.	[109]
IL-1 family (IL-1, IL-18, IL-33, IL-36, IL-37, IL-38)	Members of the IL-1 family collectively contribute to hyper-inflammatory responses in COVID-19, underpinning severe disease inflammatory pathology.	Mechanistic and exploratory study; no statistical modeling; no predictive AUC reported; No validated cut-off values were defined; integrates evidence from experimental and clinical studies	[110]
IL-6, IL-10, IL-18	These ILs showed consistently elevated levels in COVID-19 across multiple SARS-CoV-2 variants (Wuhan, Alpha, Delta, Omicron), indicating their role as variant-independent markers of infection and inflammation.	Cross-sectional, comparative analytical study; No multivariable regression models; ROC curve analyses were not performed; No cytokine cut-off thresholds were defined; Non-parametric tests (mainly Mann–Whitney U and Kruskal–Wallis tests) to compare cytokine levels between variant groups.	[34]
IL-1α, IL-4, IL-9, IL-16	Levels of these ILs were significantly higher in Delta vs. Omicron infections, suggesting variant-specific differences in combined IL responses related to immune activation intensity.	Comparative cross-sectional study; No multivariable regression models; ROC curve analyses were not performed; No cytokine cut-off thresholds were defined	[98]
IL-4 + IL-10	Both ILs are anti-inflammatory and found elevated in early Omicron	Comparative cross-sectional study; No multivariable regression models; no AUC analysis; No cytokine cut-off thresholds were defined	[103]
Elevated IL-2, IL-4, IL-10	Omicron subvariant BA.1 ≤ BA.2		[103]

## Data Availability

No new data were created or analyzed in this study. Data sharing is not applicable to this article.

## References

[1] Kindler E., Thiel V. (2014). To sense or not to sense viral RNA—Essentials of coronavirus innate immune evasion. Curr. Opin. Microbiol..

[2] Ghanbari Naeini L., Abbasi L., Karimi F., Kokabian P., Abdi Abyaneh F., Naderi D. (2023). The Important Role of Interleukin-2 in COVID-19. J. Immunol. Res..

[3] Al-Qahtani A.A., Alhamlan F.S., Al-Qahtani A.A. (2024). Pro-Inflammatory and Anti-Inflammatory Interleukins in Infectious Diseases: A Comprehensive Review. Trop. Med. Infect. Dis..

[4] Van de Veerdonk F.L., Netea M.G. (2020). Blocking IL-1 to prevent respiratory failure in COVID-19. Crit. Care.

[5] Jamilloux Y., Henry T., Belot A., Viel S., Fauter M., El Jammal T., Walzer T., François B., Sève P. (2020). Should we stimulate or suppress immune responses in COVID-19? Cytokine and anti-cytokine interventions. Autoimmun. Rev..

[6] Martinon F., Burns K., Tschopp J. (2002). The inflammasome: A molecular platform triggering activation of inflammatory caspases and processing of proIL-beta. Mol. Cell.

[7] Wang Y., Wang J., Zheng W., Zhang J., Wang J., Jin T., Tao P., Wang Y., Liu C., Huang J. (2023). Identification of an IL-1 receptor mutation driving autoinflammation directs IL-1-targeted drug design. Immunity.

[8] Dinarello C.A. (2002). The IL-1 family and inflammatory diseases. Clin. Exp. Rheumatol..

[9] Shi J., Gao W., Shao F. (2017). Pyroptosis: Gasdermin-Mediated Programmed Necrotic Cell Death. Trends Biochem. Sci..

[10] Cavalli G., De Luca G., Campochiaro C., Della-Torre E., Ripa M., Canetti D., Oltolini C., Castiglioni B., Tassan D.C., Boffini N. (2020). Interleukin-1 blockade with high-dose anakinra in patients with COVID-19, acute respiratory distress syndrome, and hyperinflammation: A retrospective cohort study. Lancet Rheumatol..

[11] Rodríguez-Morales J., Guartazaca-Guerrero S., Rizo-Téllez S.A., Viurcos-Sanabria R., Barrón E.V., Hernández-Valencia A.F., Nava P., Escobedo G., Carrillo-Ruiz J.D., Méndez-García L.A. (2022). Blood-brain Barrier Damage is Pivotal for SARS-CoV-2 Infection to the Central Nervous System. Exp. Neurobiol..

[12] Conti P., Ronconi G., Caraffa A., Gallenga C.E., Ross R., Frydas I., Kritas S.K. (2020). Induction of pro-inflammatory cytokines (IL-1 and IL-6) and lung inflammation by Coronavirus-19 (COVI-19 or SARS-CoV-2): Anti-inflammatory strategies. J. Biol. Regul. Homeost. Agents.

[13] Tjan L.H., Furukawa K., Nagano T., Kiriu T., Nishimura M., Arii J., Hino Y., Iwata S., Nishimura Y., Mori Y. (2021). Early Differences in Cytokine Production by Severity of Coronavirus Disease 2019. J. Infect. Dis..

[14] Chang Y., Bai M., You Q. (2022). Associations between Serum Interleukins (IL-1β, IL-2, IL-4, IL-6, IL-8, and IL-10) and Disease Severity of COVID-19: A Systematic Review and Meta-Analysis. Biomed Res. Int..

[15] Abbas A.K., Lichtman A.H., Pillai S. (2018). Cytokines. Cellular and Molecular Immunology.

[16] Shi H., Wang W., Yin J., Ouyang Y., Pang L., Feng Y., Qiao L., Guo X., Shi H., Jin R. (2020). The inhibition of IL-2/IL-2R gives rise to CD8+ T cell and lymphocyte decrease through JAK1-STAT5 in critical patients with COVID-19 pneumonia. Cell Death Dis..

[17] Niu C., Liang T., Chen Y., Zhu S., Zhou L., Chen N., Qian L., Wang Y., Li M., Zhou X. (2024). SARS-CoV-2 spike protein induces the cytokine release syndrome by stimulating T cells to produce more IL-2. Front. Immunol..

[18] Moore J.B., June C.H. (2020). Cytokine release syndrome in severe COVID-19. Science.

[19] Wang X., Tang G., Liu Y., Zhang L., Chen B., Han Y., Fu Z., Wang L., Hu G., Ma Q. (2022). The role of IL-6 in coronavirus, especially in COVID-19. Front. Pharmacol..

[20] Zhu J., Pang J., Ji P., Zhong Z., Li H., Li B., Zhang J. (2021). Elevated interleukin-6 is associated with severity of COVID-19: A meta-analysis. J. Med. Virol..

[21] Udomsinprasert W., Jittikoon J., Sangroongruangsri S., Chaikledkaew U. (2021). Circulating Levels of Interleukin-6 and Interleukin-10, But Not Tumor Necrosis Factor-Alpha, as Potential Biomarkers of Severity and Mortality for COVID-19: Systematic Review with Meta-analysis. J. Clin. Immunol..

[22] Coomes E.A., Haghbayan H. (2020). Interleukin-6 in Covid-19: A systematic review and meta-analysis. Rev. Med. Virol..

[23] Zhang J., Hao Y., Ou W., Ming F., Liang G., Qian Y., Cai Q., Dong S., Hu S., Wang W. (2020). Serum interleukin-6 is an indicator for severity in 901 patients with SARS-CoV-2 infection: A cohort study. J. Transl. Med..

[24] Azaiz M.B., Jemaa A.B., Sellami W., Romdhani C., Ouslati R., Gharsallah H., Ghazouani E., Ferjani M. (2022). Deciphering the balance of IL-6/IL-10 cytokines in severe to critical COVID-19 patients. Immunobiology.

[25] Gubernatorova E.O., Gorshkova E.A., Polinova A.I., Drutskaya M.S. (2020). IL-6: Relevance for immunopathology of SARS-CoV-2. Cytokine Growth Factor Rev..

[26] Di Spigna G., Covelli B., Vargas M., Di Caprio R., Rubino V., Iacovazzo C., Napolitano F., Servillo G., Postiglione L. (2024). The Behaviour of IL-6 and Its Soluble Receptor Complex during Different Waves of the COVID-19 Pandemic. Life.

[27] Zizzo G., Tamburello A., Castelnovo L., Laria A., Mumoli N., Faggioli P.M., Stefani I., Mazzone A. (2022). Immunotherapy of COVID-19: Inside and Beyond IL-6 Signalling. Front. Immunol..

[28] Jiang J., Wang J., Yao L., Lai S., Zhang X. (2021). What do we know about IL-6 in COVID-19 so far?. Biophys. Rep..

[29] Paranga T.G., Mitu I., Pavel-Tanasa M., Rosu M.F., Miftode I.L., Constantinescu D., Obreja M., Plesca C.E., Miftode E. (2024). Cytokine Storm in COVID-19: Exploring IL-6 Signaling and Cytokine-Microbiome Interactions as Emerging Therapeutic Approaches. Int. J. Mol. Sci..

[30] Li Y.S., Ren H.C., Cao J.H. (2022). Roles of Interleukin-6-mediated immunometabolic reprogramming in COVID-19 and other viral infection-associated diseases. Int. Immunopharmacol..

[31] McGonagle D., Sharif K., O’Regan A., Bridgewood C. (2020). The Role of Cytokines including Interleukin-6 in COVID-19 induced Pneumonia and Macrophage Activation Syndrome-Like Disease. Autoimmun. Rev..

[32] ElKassar N., Gress R.E. (2010). An overview of IL-7 biology and its use in immunotherapy. J. Immunotoxicol..

[33] Barata J.T., Durum S.K., Seddon B. (2019). Flip the coin: IL-7 and IL-7R in health and disease. Nat. Immunol..

[34] Korobova Z.R., Arsentieva N.A., Liubimova N.E., Batsunov O.K., Dedkov V.G., Gladkikh A.S., Sharova A.A., Adish Z., Chernykh E.I., Kaschenko V.A. (2022). Cytokine Profiling in Different SARS-CoV-2 Genetic Variants. Int. J. Mol. Sci..

[35] Laterre P.F., François B., Collienne C., Hantson P., Jeannet R., Remy K.E., Hotchkiss R.S. (2020). Association of Interleukin 7 Immunotherapy with Lymphocyte Counts Among Patients with Severe Coronavirus Disease 2019 (COVID-19). JAMA Netw. Open.

[36] Shankar-Hari M., Francois B., Remy K.E., Gutierrez C., Pastores S., Daix T., Jeannet R., Blood J., Walton A.H., Salomao R. (2025). A randomized, double-blind, placebo-controlled trial of IL-7 in critically ill patients with COVID-19. JCI Insight.

[37] Mazer M.B., Turnbull I.R., Miles S., Blood T.M., Sadler B., Hess A., Botney M.D., Martin R.S., Bosanquet J.P., Striker D.A. (2021). Interleukin-7 Reverses Lymphopenia and Improves T-Cell Function in Coronavirus Disease 2019 Patient with Inborn Error of Toll-Like Receptor 3: A Case Report. Crit. Care Explor..

[38] Siedlecka D., Bielawska L., Ludziejewska A., Baszczuk A., Wysocka E. (2025). IL-2 and IL-7 Contribution to Immune Response: Effects of Vaccination Against COVID-19 in Adults. Viruses.

[39] Bekele Y., Sui Y., Berzofsky J.A. (2021). IL-7 in SARS-CoV-2 Infection and as a Potential Vaccine Adjuvant. Front. Immunol..

[40] Sadhu S., Dalal R., Dandotiya J., Binayke A., Singh V., Tripathy M.R., Das V., Goswami S., Kumar S., Rizvi Z.A. (2023). IL-9 aggravates SARS-CoV-2 infection and exacerbates associated airway inflammation. Nat. Commun..

[41] Calvo-Alvarez E., D’Alessandro S., Zanotta N., Basilico N., Parapini S., Signorini L., Perego F., Maina K.K., Ferrante P., Modenese A. (2024). Multiplex array analysis of circulating cytokines and chemokines in COVID-19 patients during the first wave of the SARS-CoV-2 pandemic in Milan, Italy. BMC Immunol..

[42] Ghazavi A., Ganji A., Keshavarzian N., Rabiemajd S., Mosayebi G. (2021). Cytokine profile and disease severity in patients with COVID-19. Cytokine.

[43] Cao Z., Wang J., Liu X., Liu Y., Li F., Liu M., Chiu S., Jin X. (2024). Helminth alleviates COVID-19-related cytokine storm in an IL-9-dependent way. mBio.

[44] Xiang C., Zhong G., Wang H. (2024). IL-9 plays a critical role in helminth-induced protection against COVID-19-related cytokine storms. mBio.

[45] Santos G.C., Almeida C.M., Coelho E.C., Freire R.S., Silva A.A.F., Belchior L.R., Pfrimer I.A.H. (2025). Anti-inflammatory interleukins (IL-4 and IL-10) and their relationship with the severity of COVID-19: A systematic review. Res. Soc. Dev..

[46] Dhar S.K., Vishnupriyan K., Damodar S., Gujar S., Das M. (2021). IL-6 and IL-10 as predictors of disease severity in COVID-19 patients: Results from meta-analysis and regression. Heliyon.

[47] Han H., Ma Q., Li C., Liu R., Zhao L., Wang W., Zhang P., Liu X., Gao G., Liu F. (2020). Profiling serum cytokines in COVID-19 patients reveals IL-6 and IL-10 are disease severity predictors. Emerg. Microbes Infect..

[48] Rajamanickam A., Nathella P.K., Selvaraj N., Manoj M., Thangaraj J.W.V., Muthusamy S.K., Chethrapilly Purushothaman G.K., Bhatnagar T., Ponnaiah M., Ramasamy S. (2023). Characterization of IL-10 Family of Cytokines in Acute and Convalescent COVID-19 Individuals. J. Interferon Cytokine Res..

[49] Abbood S.J.A., Anvari E., Fateh A. (2023). Association between interleukin-10 gene polymorphisms (rs1800871, rs1800872, and rs1800896) and severity of infection in different SARS-CoV-2 variants. Hum. Genom..

[50] Saraiva M., Vieira P., O’Garra A. (2020). Biology and therapeutic potential of interleukin-10. J. Exp. Med..

[51] Antoniv T.T., Ivashkiv L.B. (2006). Dysregulation of interleukin-10-dependent gene expression in rheumatoid arthritis synovial macrophages. Arthritis Rheum..

[52] Ji J.D., Tassiulas I., Park-Min K.H., Aydin A., Mecklenbrauker I., Tarakhovsky A., Pricop L., Salmon J.E., Ivashkiv L.B. (2003). Inhibition of interleukin 10 signaling after Fc receptor ligation and during rheumatoid arthritis. J. Exp. Med..

[53] Islam H., Chamberlain T.C., Mui A.L., Little J.P. (2021). Elevated Interleukin-10 Levels in COVID-19: Potentiation of Pro-Inflammatory Responses or Impaired Anti-Inflammatory Action?. Front. Immunol..

[54] Najafi-Fard S., Petruccioli E., Farroni C., Petrone L., Vanini V., Cuzzi G., Salmi A., Altera A.M.G., Navarra A., Alonzi T. (2022). Evaluation of the immunomodulatory effects of interleukin-10 on peripheral blood immune cells of COVID-19 patients: Implication for COVID-19 therapy. Front. Immunol..

[55] Shih L.J., Yang C.C., Liao M.T., Lu K.C., Hu W.C., Lin C.P. (2023). An important call: Suggestion of using IL-10 as therapeutic agent for COVID-19 with ARDS and other complications. Virulence.

[56] Hasanvand A. (2022). COVID-19 and the role of cytokines in this disease. Inflammopharmacology.

[57] Sharif-Askari F.S., Sharif-Askari N.S., Hafezi S., Mdkhana B., Alsayed H.A.H., Ansari A.W., Mahboub B., Zakeri A.M., Temsah M.H., Zahir W. (2022). Interleukin-17, a salivary biomarker for COVID-19 severity. PLoS ONE.

[58] McGeachy M.J., Cua D.J., Gaffen S.L. (2019). The IL-17 Family of Cytokines in Health and Disease. Immunity.

[59] Barnes B.J., Adrover J.M., Baxter-Stoltzfus A., Borczuk A., Cools-Lartigue J., Crawford J.M., Daßler-Plenker J., Guerci P., Huynh C., Knight J.S. (2020). Targeting potential drivers of COVID-19: Neutrophil extracellular traps. J. Exp. Med..

[60] Martonik D., Parfieniuk-Kowerda A., Rogalska M., Flisiak R. (2021). The Role of Th17 Response in COVID-19. Cells.

[61] Wilson M.S., Madala S.K., Ramalingam T.R., Gochuico B.R., Rosas I.O., Cheever A.W., Wynn T.A. (2010). Bleomycin and IL-1beta-mediated pulmonary fibrosis is IL-17A dependent. J. Exp. Med..

[62] Slaats J., Ten Oever J., van de Veerdonk F.L., Netea M.G. (2016). IL-1β/IL-6/CRP and IL-18/ferritin: Distinct Inflammatory Programs in Infections. PLoS Pathog..

[63] Nakanishi K. (2018). Unique Action of Interleukin-18 on T Cells and Other Immune Cells. Front. Immunol..

[64] Blom L., Poulsen L.K. (2012). IL-1 family members IL-18 and IL-33 upregulate the inflammatory potential of differentiated human Th1 and Th2 cultures. J. Immunol..

[65] Satış H., Özger H.S., Aysert Yıldız P., Hızel K., Gulbahar Ö., Erbaş G., Aygencel G., Guzel Tunccan O., Öztürk M.A., Dizbay M. (2021). Prognostic value of interleukin-18 and its association with other inflammatory markers and disease severity in COVID-19. Cytokine.

[66] Marino L., Criniti A., Guida S., Bucci T., Ballesio L., Suppa M., Galardo G., Vacca A., Santulli M., Angeloni A. (2023). Interleukin 18 and IL-18 BP response to Sars-CoV-2 virus infection. Clin. Exp. Med..

[67] Schooling C.M., Li M., Au Yeung S.L. (2021). Interleukin-18 and COVID-19. Epidemiol. Infect..

[68] Rodrigues T.S., de Sá K.S.G., Ishimoto A.Y., Becerra A., Oliveira S., Almeida L., Gonçalves A.V., Perucello D.B., Andrade W.A., Castro R. (2021). Inflammasomes are activated in response to SARS-CoV-2 infection and are associated with COVID-19 severity in patients. J. Exp. Med..

[69] Plassmeyer M., Alpan O., Corley M.J., Premeaux T.A., Lillard K., Coatney P., Vaziri T., Michalsky S., Pang A.P.S., Bukhari Z. (2022). Caspases and therapeutic potential of caspase inhibitors in moderate-severe SARS-CoV-2 infection and long COVID. Allergy.

[70] Theobald S.J., Simonis A., Georgomanolis T., Kreer C., Zehner M., Eisfeld H.S., Albert M.C., Chhen J., Motameny S., Erger F. (2021). Long-lived macrophage reprogramming drives spike protein-mediated inflammasome activation in COVID-19. EMBO Mol. Med..

[71] Junqueira C., Crespo Â., Ranjbar S., Ingber J., Parry B., Ravid S., de Lacerda L.B., Lewandrowski M., Clark S., Ho F. (2021). SARS-CoV-2 infects blood monocytes to activate NLRP3 and AIM2 inflammasomes, pyroptosis and cytokine release. medRxiv.

[72] Liang S., Bao C., Yang Z., Liu S., Sun Y., Cao W., Wang T., Schwantes-An T.H., Choy J.S., Naidu S. (2023). SARS-CoV-2 spike protein induces IL-18-mediated cardiopulmonary inflammation via reduced mitophagy. Signal Transduct. Target. Ther..

[73] Pierce C.A., Sy S., Galen B., Goldstein D.Y., Orner E., Keller M.J., Herold K.C., Herold B.C. (2021). Natural mucosal barriers and COVID-19 in children. JCI Insight.

[74] Williams M.A., O’Callaghan A., Corr S.C. (2019). IL-33 and IL-18 in Inflammatory Bowel Disease Etiology and Microbial Interactions. Front. Immunol..

[75] Tao W., Zhang G., Wang X., Guo M., Zeng W., Xu Z., Cao D., Pan A., Wang Y., Zhang K. (2020). Analysis of the intestinal microbiota in COVID-19 patients and its correlation with the inflammatory factor IL-18. Med. Microecol..

[76] Singh R., Hemati H., Bajpai M., Yadav P., Maheshwari A., Kumar S., Agrawal S., Sevak J.K., Islam M., Mars J.S. (2021). Sustained expression of inflammatory monocytes and activated T cells in COVID-19 patients and recovered convalescent plasma donors. Immun. Inflamm. Dis..

[77] Meka R.R., Venkatesha S.H., Dudics S., Acharya B., Moudgil K.D. (2015). IL-27-induced modulation of autoimmunity and its therapeutic potential. Autoimmun. Rev..

[78] Valdés-López J.F., Urcuqui-Inchima S. (2023). Antiviral response and immunopathogenesis of interleukin 27 in COVID-19. Arch. Virol..

[79] Hamdy H., Elhamammy R.H., Abdelmageed M., Wahid A. (2025). Impact of single nucleotide polymorphism of IL-27P28 rs153109 and IFITM3 rs12252 on susceptibility and severity of COVID-19 in Egyptian patients: A case control study. Virol. J..

[80] Korobova Z.R., Arsentieva N.A., Santoni A., Totolian A.A. (2024). Role of IL-27 in COVID-19: A Thin Line between Protection and Disease Promotion. Int. J. Mol. Sci..

[81] Waseem M., Shariff M.A., Lim C.A., Nunez J., Narayanan N., Patel K., Tay E.T. (2022). Multisystem Inflammatory Syndrome in Children. West. J. Emerg. Med..

[82] Spracklen T.F., Mendelsohn S.C., Butters C., Facey-Thomas H., Stander R., Abrahams D., Erasmus M., Baguma R., Day J., Scott C. (2022). IL27 gene expression distinguishes multisystem inflammatory syndrome in children from febrile illness in a South African cohort. Front. Immunol..

[83] Liu Q., Dwyer G.K., Zhao Y., Li H., Mathews L.R., Chakka A.B., Chandran U.R., Demetris J.A., Alcorn J.F., Robinson K.M. (2019). IL-33-mediated IL-13 secretion by ST2+ Tregs controls inflammation after lung injury. JCI Insight.

[84] Xu J., Guardado J., Hoffman R., Xu H., Namas R., Vodovotz Y., Xu L., Ramadan M., Brown J., Turnquist H.R. (2017). IL33-mediated ILC2 activation and neutrophil IL5 production in the lung response after severe trauma: A reverse translation study from a human cohort to a mouse trauma model. PLoS Med..

[85] Burke H., Freeman A., Cellura D.C., Stuart B.L., Brendish N.J., Poole S., Borca F., Phan H.T.T., Sheard N., Williams S. (2020). Inflammatory phenotyping predicts clinical outcome in COVID-19. Respir. Res..

[86] Fonseca W., Asai N., Yagi K., Malinczak C.A., Savickas G., Johnson C.C., Murray S., Zoratti E.M., Lukacs N.W., Li J. (2021). COVID-19 Modulates Inflammatory and Renal Markers That May Predict Hospital Outcomes among African American Males. Viruses.

[87] Gao Y., Cai L., Li L., Zhang Y., Li J., Luo C., Wang Y., Tao L. (2022). Emerging Effects of IL-33 on COVID-19. Int. J. Mol. Sci..

[88] Stanczak M.A., Sanin D.E., Apostolova P., Nerz G., Lampaki D., Hofmann M., Steinmann D., Krohn-Grimberghe M., Thimme R., Mittler G. (2021). IL-33 expression in response to SARS-CoV-2 correlates with seropositivity in COVID-19 convalescent individuals. Nat. Commun..

[89] Retnakumar S.V., Singh S.C., Chauvin C., Bayry J. (2025). IL-33 and IL-3 synergistically induce CD25 expression on human basophils without functional IL-2 signaling: A potential marker of severe COVID-19. Front. Immunol..

[90] Wang X., Yi P., Liang Y. (2021). The Role of IL-36 in Infectious Diseases: Potential Target for COVID-19?. Front. Immunol..

[91] Monteil V., Kwon H., Prado P., Hagelkrüys A., Wimmer R.A., Stahl M., Leopoldi A., Garreta E., Hurtado Del Pozo C., Prosper F. (2020). Inhibition of SARS-CoV-2 Infections in Engineered Human Tissues Using Clinical-Grade Soluble Human ACE2. Cell.

[92] Zulli A., Burrell L.M., Buxton B.F., Hare D.L. (2008). ACE2 and AT4R are present in diseased human blood vessels. Eur. J. Histochem..

[93] Mercurio L., Failla C.M., Capriotti L., Scarponi C., Facchiano F., Morelli M., Rossi S., Pagnanelli G., Albanesi C., Cavani A. (2020). Interleukin (IL)-17/IL-36 axis participates to the crosstalk between endothelial cells and keratinocytes during inflammatory skin responses. PLoS ONE.

[94] Joo Y.H., Kim H.K., Hak Choi I., Han H.M., Lee K.J., Kim T.H., Lee S.H. (2020). Increased expression of interleukin 36 in chronic rhinosinusitis and its contribution to chemokine secretion and increased epithelial permeability. Cytokine.

[95] National Center for Immunization and Respiratory Diseases (U.S.) Division of Viral Diseases. SARS-CoV-2 Variant Classifications and Definitions. https://stacks.cdc.gov/view/cdc/133705.

[96] Bostanghadiri N., Ziaeefar P., Mofrad M.G., Yousefzadeh P., Hashemi A., Darban-Sarokhalil D. (2023). COVID-19: An Overview of SARS-CoV-2 Variants-The Current Vaccines and Drug Development. Biomed Res. Int..

[97] Raman R., Patel K.J., Ranjan K. (2021). COVID-19: Unmasking Emerging SARS-CoV-2 Variants, Vaccines and Therapeutic Strategies. Biomolecules.

[98] Krivosova M., Hanusrichterova J., Lucansky V., Samec M., Bobcakova A., Baranovicova E., Dohál M., Mokry J., Škereňová M., Lipták P. (2025). Comparative study of cytokine profiles in SARS-CoV-2 Delta and Omicron variants. Bratisl. Med. J..

[99] Merchant M., Ashraf J., Masood K.I., Yameen M., Hussain R., Nasir A., Hasan Z. (2024). SARS-CoV-2 variants induce increased inflammatory gene expression but reduced interferon responses and heme synthesis as compared with wild type strains. Sci. Rep..

[100] Li X., Li W., Liu Z., Kang Y., Zhang X., Xu Z., Gao Y., Qi Y. (2022). A comparative study of spike protein of SARS-CoV-2 and its variant Omicron (B.1.1.529) on some immune characteristics. Sci. Rep..

[101] Shahbaz S., Bozorgmehr N., Lu J., Osman M., Sligl W., Tyrrell D.L., Elahi S. (2023). Analysis of SARS-CoV-2 isolates, namely the Wuhan strain, Delta variant, and Omicron variant, identifies differential immune profiles. Microbiol. Spectr..

[102] Li F., Wang Y., Jin M., Li H., Yan J., Hu J., Zhang X., Wu C., Wei L. (2024). A comprehensive analysis of immune characteristics and clinical prognosis in Asian COVID-19 patients infected with SARS-CoV-2 Omicron strain XBB sub-variants: A retrospective study of 450 cases. Arch. Med. Sci..

[103] Barreto M.A., Cruz A.M.S., Volle D.M., Júnior W.D.D.C., Costa I.B., Nunes J.A.L., de Sousa A.C.P., Lima I.K.M., Nogami P.Y., Borges I.R. (2025). Clinical Manifestations and Cytokine Profiles of the Th1, Th2, and Th17 Response Associated with SARS-CoV-2 Omicron Subvariants. Biomedicines.

[104] Reuschl A.K., Thorne L.G., Whelan M.V.X., Ragazzini R., Furnon W., Cowton V.M., De Lorenzo G., Mesner D., Turner J.L.E., Dowgier G. (2024). Evolution of enhanced innate immune suppression by SARS-CoV-2 Omicron subvariants. Nat. Microbiol..

[105] Liu Y., Guo Y., Zhan H., Liu X., Li X., Cui J., Li H., Feng S., Cheng L., Li X. (2024). Immune and inflammation features of severe and critical Omicron infected patients during Omicron wave in China. BMC Infect. Dis..

[106] Irvem A., Erdogan Cakir D., Aydın C., Mart Komurcu S.Z., Celik S., Kazezoğlu C. (2025). Effect of SARS-CoV-2 variants (Alpha, Beta, Delta, Omicron) on inflammatory parameters. Jundishapur J. Microbiol..

[107] Koutsakos M., Lee W.S., Wheatley A.K., Kent S.J., Juno J.A. (2022). T follicular helper cells in the humoral immune response to SARS-CoV-2 infection and vaccination. J. Leukoc. Biol..

[108] Kawasuji H., Morinaga Y., Nagaoka K., Tani H., Yoshida Y., Yamada H., Takegoshi Y., Kaneda M., Murai Y., Kimoto K. (2024). High interleukin-6 levels induced by COVID-19 pneumonia correlate with increased circulating follicular helper T cell frequency and strong neutralization antibody response in the acute phase of Omicron breakthrough infection. Front. Immunol..

[109] Ghofrani Nezhad M., Jami G., Kooshkaki O., Chamani S., Naghizadeh A. (2023). The Role of Inflammatory Cytokines (Interleukin-1 and Interleukin-6) as a Potential Biomarker in the Different Stages of COVID-19 (Mild, Severe, and Critical). J. Interferon Cytokine Res..

[110] Makaremi S., Asgarzadeh A., Kianfar H., Mohammadnia A., Asghariazar V., Safarzadeh E. (2022). The role of IL-1 family of cytokines and receptors in pathogenesis of COVID-19. Inflamm. Res..

[111] Lariccia V., Magi S., Serfilippi T., Toujani M., Gratteri S., Amoroso S. (2020). Challenges and Opportunities from Targeting Inflammatory Responses to SARS-CoV-2 Infection: A Narrative Review. J. Clin. Med..

[112] Davidson M., Menon S., Chaimani A., Evrenoglou T., Ghosn L., Graña C., Henschke N., Cogo E., Villanueva G., Ferrand G. (2022). Interleukin-1 blocking agents for treating COVID-19. Cochrane Database Syst. Rev..

[113] Landi L., Ravaglia C., Russo E., Cataleta P., Fusari M., Boschi A., Giannarelli D., Facondini F., Valentini I., Panzini I. (2020). Blockage of interleukin-1β with canakinumab in patients with Covid-19. Sci. Rep..

[114] Liang P., Li Y., Meng L., Li Y., Mai H., Li T., Ma J., Ma J., Wang J., Zhuan B. (2024). Prognostic significance of serum interleukin-6 in severe/critical COVID-19 patients treated with tocilizumab: A detailed observational study analysis. Sci. Rep..

[115] World Health Organization WHO Prequalifies First Monoclonal Antibody—Tocilizumab—To Treat COVID-19.

[116] Ghosn L., Assi R., Evrenoglou T., Buckley B.S., Henschke N., Probyn K., Riveros C., Davidson M., Graña C., Bonnet H. (2023). Interleukin-6 blocking agents for treating COVID-19: A living systematic review. Cochrane Database Syst. Rev..

[117] Han Q., Guo M., Zheng Y., Zhang Y., De Y., Xu C., Zhang L., Sun R., Lv Y., Liang Y. (2020). Current Evidence of Interleukin-6 Signaling Inhibitors in Patients with COVID-19: A Systematic Review and Meta-Analysis. Front. Pharmacol..

[118] Rahimi F., Bezmin Abadi A.T. (2022). WHO prequalified tocilizumab and vaccine boosters against COVID-19. Int. J. Surg..

[119] Potere N., Batticciotto A., Vecchié A., Porreca E., Cappelli A., Abbate A., Dentali F., Bonaventura A. (2021). The role of IL-6 and IL-6 blockade in COVID-19. Expert. Rev. Clin. Immunol..

[120] Thys K., Loza M.J., Lynn L., Callewaert K., Varma L., Crabbe M., Van Wesenbeeck L., Van Landuyt E., De Meyer S., Aerssens J. (2024). Pharmacodynamic, prognostic, and predictive biomarkers in severe and critical COVID-19 patients treated with sirukumab. Sci. Rep..

[121] Fang S., Ju D., Lin Y., Chen W. (2022). The role of interleukin-22 in lung health and its therapeutic potential for COVID-19. Front. Immunol..

[122] Albayrak N., Orte Cano C., Karimi S., Dogahe D., Van Praet A., Godefroid A., Del Marmol V., Grimaldi D., Bondue B., Van Vooren J.P. (2022). Distinct Expression Patterns of Interleukin-22 Receptor 1 on Blood Hematopoietic Cells in SARS-CoV-2 Infection. Front. Immunol..

[123] Klooster J.P.T., Bol-Schoenmakers M., van Summeren K., van Vliet A.L.W., de Haan C.A.M., van Kuppeveld F.J.M., Verkoeijen S., Pieters R. (2021). Enterocytes, fibroblasts and myeloid cells synergize in anti-bacterial and anti-viral pathways with IL22 as the central cytokine. Commun. Biol..

[124] García M., Kokkinou E., Carrasco García A., Parrot T., Palma Medina L.M., Maleki K.T., Christ W., Varnaitė R., Filipovic I., Ljunggren H.G. (2020). Innate lymphoid cell composition associates with COVID-19 disease severity. Clin. Transl. Immunol..

[125] Alcorn J.F. (2020). IL-22 Plays a Critical Role in Maintaining Epithelial Integrity During Pulmonary Infection. Front. Immunol..

[126] Pociask D.A., Scheller E.V., Mandalapu S., McHugh K.J., Enelow R.I., Fattman C.L., Kolls J.K., Alcorn J.F. (2013). IL-22 is essential for lung epithelial repair following influenza infection. Am. J. Pathol..

[127] Bhaumik S., Basu R. (2017). Cellular and Molecular Dynamics of Th17 Differentiation and its Developmental Plasticity in the Intestinal Immune Response. Front. Immunol..

[128] Wu D., Yang X.O. (2020). TH17 responses in cytokine storm of COVID-19: An emerging target of JAK2 inhibitor Fedratinib. J. Microbiol. Immunol. Infect..

[129] Mendoza V.M.M. (2020). Interleukin-17: A potential therapeutic target in COVID-19. J. Infect..

[130] Resende G.G., da Cruz Lage R., Lobê S.Q., Medeiros A.F., Costa ESilva A.D., Nogueira Sá A.T., Oliveira A.J.A., Sousa D., Guimarães H.C., Gomes I.C. (2022). Blockade of interleukin seventeen (IL-17A) with secukinumab in hospitalized COVID-19 patients—The BISHOP study. Infect. Dis..

[131] Avdeev S.N., Trushenko N.V., Tsareva N.A., Yaroshetskiy A.I., Merzhoeva Z.M., Nuralieva G.S., Nekludova G.V., Chikina S.Y., Gneusheva T.Y., Suvorova O.A. (2021). Anti-IL-17 monoclonal antibodies in hospitalized patients with severe COVID-19: A pilot study. Cytokine.

[132] Stebbing J., Krishnan V., de Bono S., Ottaviani S., Casalini G., Richardson P.J., Monteil V., Lauschke V.M., Mirazimi A., Youhanna S. (2020). Mechanism of baricitinib supports artificial intelligence-predicted testing in COVID-19 patients. EMBO Mol. Med..

[133] Orlov M., Wander P.L., Morrell E.D., Mikacenic C., Wurfel M.M. (2020). A Case for Targeting Th17 Cells and IL-17A in SARS-CoV-2 Infections. J. Immunol..

[134] Kakugawa T., Mimura Y., Mimura-Kimura Y., Doi K., Ohteru Y., Kakugawa H., Oishi K., Kakugawa M., Hirano T., Matsunaga K. (2024). Kinetics of pro- and anti-inflammatory spike-specific cellular immune responses in long-term care facility residents after COVID-19 mRNA primary and booster vaccination: A prospective longitudinal study in Japan. Immun. Ageing.

[135] Hurme A., Jalkanen P., Heroum J., Liedes O., Vara S., Melin M., Teräsjärvi J., He Q., Pöysti S., Hänninen A. (2022). Long-Lasting T Cell Responses in BNT162b2 COVID-19 mRNA Vaccinees and COVID-19 Convalescent Patients. Front. Immunol..

[136] Bergamaschi C., Terpos E., Rosati M., Angel M., Bear J., Stellas D., Karaliota S., Apostolakou F., Bagratuni T., Patseas D. (2021). Systemic IL-15, IFN-γ, and IP-10/CXCL10 signature associated with effective immune response to SARS-CoV-2 in BNT162b2 mRNA vaccine recipients. Cell Rep..

[137] Malahe S.R.K., Hartog Y.D., Rietdijk W.J.R., van Baarle D., de Kuiper R., Reijerkerk D., Ras A.M., Geers D., Diavatopoulos D.A., Messchendorp A.L. (2023). The role of interleukin-21 in COVID-19 vaccine-induced B cell-mediated immune responses in patients with kidney disease and kidney transplant recipients. Am. J. Transplant..

